# Cultured Cortical Neurons Can Perform Blind Source Separation According to the Free-Energy Principle

**DOI:** 10.1371/journal.pcbi.1004643

**Published:** 2015-12-21

**Authors:** Takuya Isomura, Kiyoshi Kotani, Yasuhiko Jimbo

**Affiliations:** 1 Department of Human and Engineered Environmental Studies, Graduate School of Frontier Sciences, The University of Tokyo, Hongo, Bunkyo-ku, Tokyo, Japan; 2 Research Fellow of Japan Society for the Promotion of Science (JSPS), Kojimachi, Chiyoda-ku, Tokyo, Japan; 3 Research Center for Advanced Science and Technology, The University of Tokyo, Komaba, Meguro-ku, Tokyo, Japan; 4 PRESTO, Japan Science and Technology Agency, Honcho, Kawaguchi-shi, Saitama, Japan; 5 Department of Precision Engineering, School of Engineering, The University of Tokyo, Hongo, Bunkyo-ku, Tokyo, Japan; Duke University, UNITED STATES

## Abstract

Blind source separation is the computation underlying the cocktail party effect––a partygoer can distinguish a particular talker’s voice from the ambient noise. Early studies indicated that the brain might use blind source separation as a signal processing strategy for sensory perception and numerous mathematical models have been proposed; however, it remains unclear how the neural networks extract particular sources from a complex mixture of inputs. We discovered that neurons in cultures of dissociated rat cortical cells could learn to represent particular sources while filtering out other signals. Specifically, the distinct classes of neurons in the culture learned to respond to the distinct sources after repeating training stimulation. Moreover, the neural network structures changed to reduce free energy, as predicted by the free-energy principle, a candidate unified theory of learning and memory, and by Jaynes’ principle of maximum entropy. This implicit learning can only be explained by some form of Hebbian plasticity. These results are the first *in vitro* (as opposed to *in silico*) demonstration of neural networks performing blind source separation, and the first formal demonstration of neuronal self-organization under the free energy principle.

## Author Summary

The ‘cocktail party’ effect is a phenomenon by which one is able to pick out and listen to a single person’s speech in a noisy room. In information engineering, this is termed blind source separation. Numerous computational studies demonstrate that simulated neural networks can perform blind source separation. However, if or how a living neural network learns to perform blind source separation remains unknown. Using a microelectrode array (MEA) system that allowed us to apply composite inputs and record responses from neurons throughout a cultured neural network, we discovered that even neurons in cultures of dissociated rat cortical cells can separate individual sources from a complex mixture of inputs in the absence of teacher signals. Given these findings, we then determined that the neural networks adapted to reduce free energy, as predicted by the free energy principle and Jaynes’ principle of maximum entropy. These results provide evidence that cultured neural networks can perform blind source separation and that they are governed by the free-energy principle, providing a compelling framework for understanding how the brain identifies and processes signals hidden in complex multivariate information.

## Introduction

Blind source separation is a problem of separating independent sources from a complex mixture of inputs without knowledge about sources [1–4] and is the computation underlying the cocktail party effect––a phenomenon by which one is able to listen to a single person’s speech in a noisy room [5–8]. Understanding the basis of blind source separation, as well as other learning and memory processes, requires characterization of the underlying functional network architecture. Presumably, this can be directly accomplished by measuring the activity of individual neurons during blind source separation processing to establish the role of each neuron in the network. In practice, this is enormously challenging, given both the large number of neurons that may reside in a network and the technical limitations encountered in attempting to distinguish the activity of neurons that perform blind source separation from others throughout the network. As a result, most studies of blind source separation rely on simulations and on computational models, and the possible electrophysiological basis for any such information processing in real neurons remains poorly understood.

Theoretically, blind source separation is classed as unsupervised learning, a type of learning that does not require teacher signals [9–11]. Blind source separation is modeled as principal component analysis (PCA) [12], as independent component analysis (ICA) [13, 14], or as sparse coding [15, 16]. These are widely used for signal processing where separation of sources from a complex mixture of inputs is desired. Neural network models that include neurons with linear firing rates can perform PCA, a model that describes how neurons in artificial networks can strengthen or weaken their interconnections over time [12]. In contrast, ICA, which can be represented using model neurons with nonlinear firing rates [13, 14], maximizes Shannon entropy among outputs in order to detect several independent sources, thus separating a multivariate signal into individual components. The sparse coding model detects independent sources [15, 16] using a calculation similar to that proposed by the predictive coding hypothesis of the cerebral cortex [17]. What all these models of unsupervised learning have in common is that they can be implemented with a form of Hebbian or associative plasticity [18] and that they are instances of the free energy principle––a candidate unified theory of learning and memory [19, 20]. Moreover, blind source separation, whether by PCA, ICA, or sparse coding, is one of the simplest problems that the free-energy principle addresses. Additionally, numerous computational studies have demonstrated that simulated neural networks can perform blind source separation. PCA, ICA, and sparse coding have been demonstrated in both firing-rate models and spiking-neuron models [21–27]. However, although early studies indicated that cortical neurons might use an ICA-like signal processing strategy for sensory perception [5–8] and described the relationship of sparse- and predictive coding to biological properties [28, 29], examinations of the neural basis of ICA-like learning are few.

Experimental studies on *in vivo* or *in vitro* networks have demonstrated that neural networks can perform learning and memory tasks, when learning is defined as the process of changing activity or behavior by experiencing something, as it is in this study. One of the simplest networks can be constructed from actual cultured neurons, and such real neural networks can exhibit stimulation-dependent synaptic plasticity [30, 31], supervised learning [32], adaptation to inputs [33], associative memory [34], aspects of logical operation [35, 36], short-term memory [37], and homeostatic plasticity [38, 39]. However, it is uncertain whether these biological neural networks can perform blind source separation. Previously, we have used the microelectrode array (MEA) to simultaneously stimulate and record from multiple neurons over long periods [30, 40]. The MEA enables random electrical stimulation from 64 electrodes in parallel and the recording of evoked spikes immediately after each stimulation. Thus, by varying probabilities during stimulation trains, the MEA makes it possible to apply spatiotemporal inputs synthesized from hidden sources while measuring the response evoked from the entire neural network. Through this capability, we demonstrate here that cultured rat cortical neurons receiving multiple inputs can perform blind source separation, thereby providing an *in vitro* model of neural adaptation.

In brief, our approach consisted of two parts. First, we tried to establish whether single neuron responses preferred mixtures of sources or the individual sources per se. To address this, we examined the Kullback-Leibler divergence [11] between the probabilities of neuronal responses conditioned upon one of two sources. We hoped to see that neurons were able to discriminate between sources rather than mixtures, because this would imply a blind source separation––or the inversion of a generative model of stimulation patterns (i.e., sources). We were able to show that neurons preferred hidden sources, as opposed to mixtures of sources. This then allowed us to quantify the probabilistic encoding of sources by assuming that the expected amplitude of each hidden source was encoded by the mean activity of neuronal populations preferring one source or the other. By assuming a rate coding model, where mean firing rates encode the mean of a mixture of Gaussians, we were able to compute the variational free energy of the neuronal encodings in terms of energy and entropy. Crucially, the free energy principle suggests that with learning, energy should decrease and entropy should increase (where the free energy is the difference) [19, 20]. In this instance, the energy can be thought of as level of prediction error. Conversely, the entropy refers to the average uncertainty of the encoding. According to Jaynes’ maximum entropy principle [41, 42], entropy should increase to ensure a generalizable inference that is in accordance with Occam’s principle. In short, we hoped to see an increase in the entropy of the probabilistic encoding that was offset by a decrease in energy (an increase in accuracy)––producing an overall decrease in free energy.

## Results

### Generation and definition of neural stimuli

**Fig 1:**
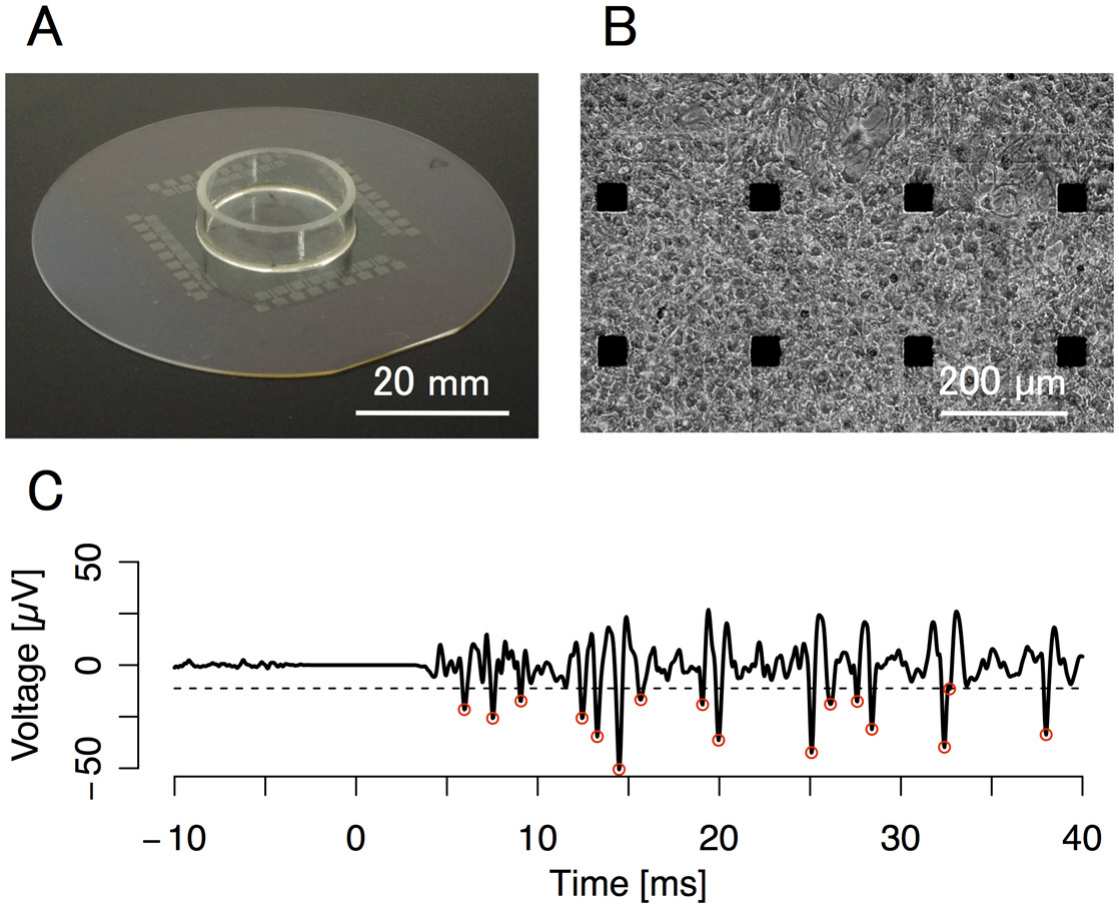
Description of the cultured neuron network paradigm. Panels show culture dish (top left), microscopic view of rat cortical cells (top right), and evoked voltage trace from one electrode (bottom). **(A)** Image of a microelectrode array (MEA) dish. Cells were seeded in the center of the MEA dishes. The microelectrode sampling frequency was 25 kHz and a 500–2000 Hz band-pass filter was applied to the recordings. **(B)** Phase-contrast microscopic images of cultured rat cortical cells on MEA dishes after 52 days in culture. Note the high concentration of cells near the electrode terminals. Black squares are electrodes. **(C)** A typical waveform of the extracellular potential. After a biphasic-pulse electrical stimulation (*τ* = 0), several stimulation-evoked spikes were observed at the stimulated electrode. The dashed line indicates the detection threshold. Red circles indicate detected spikes.

Rat cortical cells were cultured on MEAs (Fig 1A and 1B) and electrical stimulation and recordings were conducted. Typical stimulus-evoked responses of cultured neural networks recorded at the stimulated electrode are shown in Fig 1C. In accordance with previous studies, we observed tri-phasic responses [30, 40].

**Fig 2:**
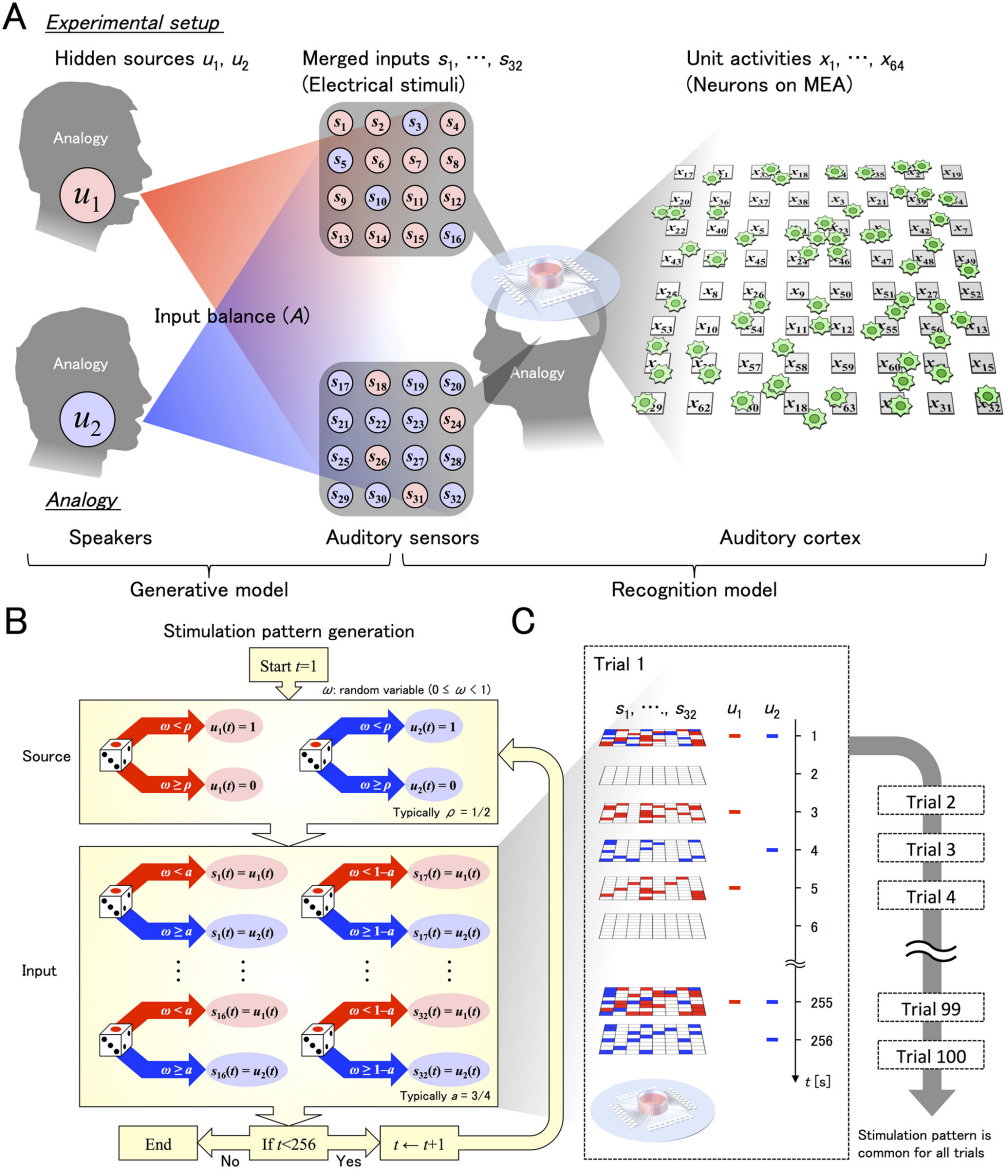
Schematic images of experimental protocol. The signal distribution scheme (top), signal generation protocol (bottom left), and training timeline (bottom right) are shown. **(A)** A schematic image of stimulation signals and neurons on an MEA. *u*_1_ and *u*_2_ are hidden sources (left), *s*_1_, …, *s*_16_ and *s*_17_, …, *s*_32_ are two groups of inputs synthesized by computing weighted combinations of the sources (middle), and *x*_1_, …, *x*_64_ are the measures at the electrodes (right). By manipulating the inputs to the MEA, we induced an ICA-like processing system in neuronal cultures. Specifically, we created four conditions of stimulation by independently varying the probabilities of two binary hidden signals (given by a 2 × 2 matrix) such that neurons received no signal (0,0), a signal in which the two hidden signals (0,1) or (1,0) were differentially weighted, or a fully merged signal (1,1). The schematic heads illustrate the analogy between the experimental setup and the cocktail party effect: *u*_1_ and *u*_2_ are analogous to two voices to identify (left), the mapping from **u** to **s** (a matrix *A*) is analogous to the addition of background noise at a cocktail party, so that **s** represents sounds (sensory inputs) heard from the left and right ears (middle), and **x** is analogous to the listener’s auditory system performing blind source separation (right). **(B)** A schematic image of the generation of **s**(*t*) from *u*_1_(*t*) and *u*_2_(*t*). Each random variable *ω* is independently generated from a uniform distribution (0 ≤ *ω* < 1). *u*_1_(*t*) and *u*_2_(*t*) will be 1 if *ω* < *ρ* or 0 otherwise. Then, *s*_1_(*t*), …, *s*_16_(*t*) will be *u*_1_(*t*) if *ω* < *a* or *u*_2_(*t*) otherwise. In contrast, *s*_17_(*t*), …, *s*_32_(*t*) will be *u*_1_(*t*) if *ω* < 1–*a* or *u*_2_(*t*) otherwise. The discrete time *t* is over one and 256. **(C)** A training timeline. As electrodes on the MEA are distributed as an 8 × 8 matrix, we illustrate the stimulating sites corresponding to *s*_1_(*t*), …, *s*_32_(*t*) on 8 × 8 matrices. Thus, half (32) were dual-use electrodes of stimulating and recording, while the remaining 32 were for recording only. Red or blue squares indicate the electrode stimulated in a given time period, which is provided from *u*_1_(*t*) or *u*_2_(*t*), respectively. A trial is composed of 256 stimulation patterns with 1-s intervals. Overall, the training period is composed of 100 trials, where the stimulation pattern is common for all trials.

To study ICA-like learning in networks created in these neuronal cultures, we designed a generative process constructed from two independent binary sources **u**(*t*) = (*u*_1_(*t*), *u*_2_(*t*))^*T*^ ∈ {0,1}, 32 inputs produced by the MEA **s**(*t*) = (*s*_1_(*t*), …, *s*_32_(*t*))^*T*^, and a 32×2 matrix *A*, where (*A*_i1_, *A*_i2_) = (*a*, 1–*a*) for *i* = 1, …, 16 and (*A*_i1_, *A*_i2_) = (1–*a*, *a*) for *i* = 17, …, 32 (Fig 2A). Note that *t* [s] is discrete time (a natural number) between 1 and 256.

In brief, we had an array of (8×8) 64 recording electrode sites of which half (32) were stimulated. The remaining 32 were for recording neural activities at non-stimulated electrodes. The detailed neural response properties are discussed in the next section. The stimuli were formed by mixing two underlying patterns, or hidden sources, to create stochastic stimulus patterns. These were mixed separately for each of two groups of 16 stimulation electrodes, such that the stimulation pattern comprised of probabilistic mixtures of the underlying sources. The responses from the 64 electrodes and 23 cultures were pooled, yielding over 1000 electrode responses to various mixtures of hidden sources.

In other words, **u**(*t*) was generated from the stationary Poisson process, while **s**(*t*) obeyed the non-stationary Poisson process with the time varying intensity of *A* **u**(*t*). The generative model ensured that the two sources contributed to the stimuli with an equal probability *ρ*. We used mixtures of these sources to produce stimulus patterns that contained no signal, one of the two sources, and a fully mixed source. Unless specifically mentioned, we used *ρ* = 1/2 and *a* = 3/4. Electrical stimulations with 256-s pulse trains were applied at 1-s intervals for 100 trials. A schematic image of how inputs **s**(*t*) were obtained from sources **u**(*t*) is shown in Fig 2B and 2C. A detailed description is provided in the Fig 2 legend and the Methods section.

### Evoked responses show preferences to individual stimuli

**Fig 3:**
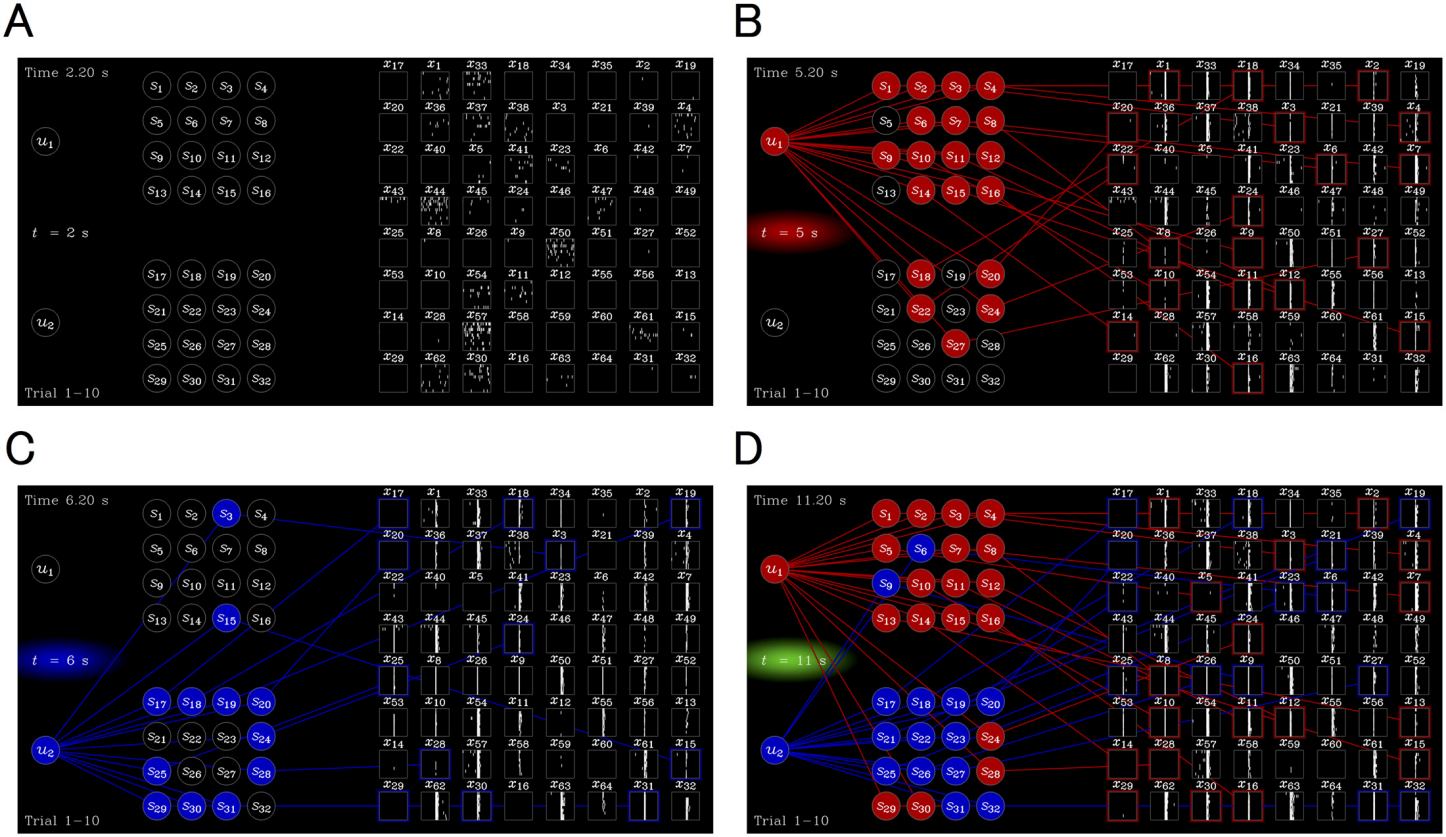
Overview of the experiment. Snapshots of S1 Movie at the **u** = (0,0), (1,0), (0,1), and (1,1) state, respectively, are shown. Setup is with the same as that described in Fig 2A: hidden sources *u*_1_ and *u*_2_ (left), merged inputs *s*_1_, …, *s*_16_ (middle top), *s*_17_, …, *s*_32_ (middle bottom), and unit activities of cultured neurons *x*_1_, …, *x*_64_ (right). Each of the 64 panels on the right shows a raster plot of neural activity (unit activity) recorded at the electrode, where horizontal and vertical axes are 400 ms time window and trials 1–10, respectively. **(A)** When **u** = (0,0), evoked responses were not observed since there was no input, although spontaneous activities were recorded. **(B)** When **u** = (1,0), a group of *s*_1_, …, *s*_16_, became 1 (red circles in the middle) with a high probability (namely, *a* = 3/4 probability), while a group of *s*_17_, …, *s*_32_ became 1 with a low probability (1–*a* = 1/4). If *s*_*i*_ (*i* = 1, …, 32) was 1, an electrical pulse stimulation was induced into a fixed corresponding electrode. Consequently, evoked responses were observed immediately after each stimulation. **(C)** When **u** = (0,1), a situation exactly opposite to that described in (B) occurs. **(D)** When u = (1,1), all stimulated electrodes (*s*_1_, …, *s*_32_) were stimulated, providing the largest evoked response.

**Fig 4:**
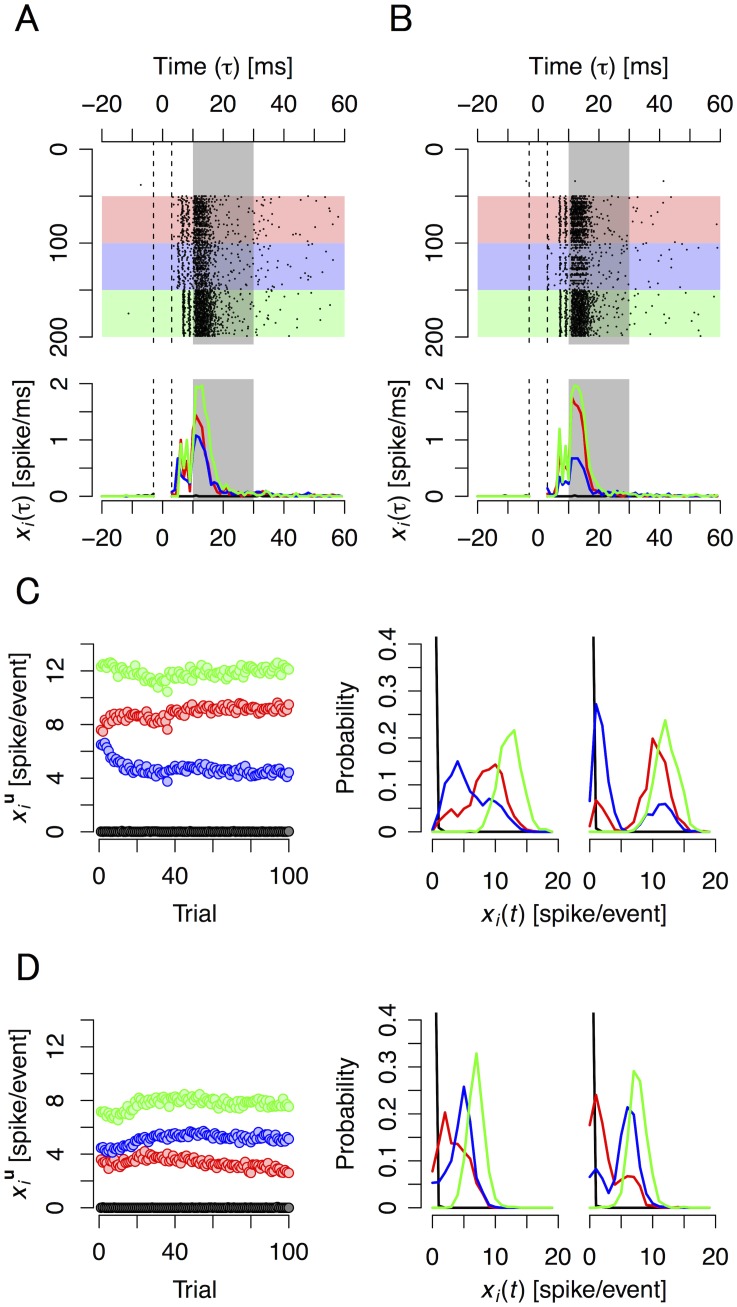
Examples of neural responses to different stimulus inputs. Raster plots of successive evoked responses before and after learning (A and B) for four source states; spikes-per-event transition over learning, average and histogram, for four source states and two different electrodes (C and D). **(A)** Top panel: A raster plot showing a typical pattern of stimulation-evoked spikes in cultured neurons recorded with an electrode at trial 1 (before training). Red circles indicate the timing of spikes. The horizontal axis corresponds to time (ms), and the vertical axis is the stimulation number sorted by source state. White, red, blue, and green areas indicate responses when the state of the source was **u** = (0,0), (1,0), (0,1), and (1,1), respectively. Between *τ* = –3 and 3 ms (area surrounded by dashed lines), reliable data were not obtained because of switching noise (artifact). Bottom panel: Post stimulus time histogram (PSTH) at trial 1. Black, red, blue, and green curves are PSTH when the state of the source was **u** = (0,0), (1,0), (0,1), and (1,1), respectively. **(B)** Same as (A), but at trial 100 (after training). **(C)** Left panel: example of a typical transition over trials of the conditional expectation of an evoked response recorded with the same electrode as in (A) and (B). Black, red, blue, and green circles give the conditional expectation of evoked responses when the state of the source was **u** = (0,0), (1,0), (0,1), and (1,1), respectively. Center and right panels: The conditional probability distributions of evoked responses recorded with the same electrode during trials 1 to 10 (center panel) and trials 91 to 100 (right panel). The four curves correspond to the four states of **u**. **(D)** Same as (C), but recorded with a different electrode.

Neural responses evoked by the input trains were recorded using a 64-electrode MEA. We used 23 cultures for a training group and a total of 37 cultures as control groups. We performed 100 trials (500 s for 1 trial; about 14 h in total) for each culture. An overview of the experimental paradigm is shown in Fig 3 and S1 and S2 Movies. A raster plot and post stimulus time histogram (PSTH) detailing the spike timing of evoked response recorded at a representative electrode (*x*_*i*_(*τ*); *τ*, continuous time) is shown in Fig 4A and 4B. Evoked response increased immediately after each stimulation for both stimulated and non-stimulated neuron groups. The peak of evoked responses was observed 10-to-20 ms after each stimulation in all trials. Compared to the results of the first trial (Fig 4A), the evoked response for the hidden source of **u** = (0,1) (blue curve) decreased after the training stimulation (Fig 4B), indicating that neurons recorded at this electrode tuned their activity to only respond to the (1,0) and (1,1) states, i.e., only to *u*_1_.

According to previous studies, the directly evoked responses occur immediately after stimulation and their jitters are relatively small; thus, large numbers of spikes that appear more than 10 ms after stimulation are generated by synaptic inputs [43]. Therefore, the change in number of evoked spikes generated 10–30 ms after each stimulation, defined as evoked response, occurred gradually over training (Fig 4C left). The center and right panels in Fig 4C illustrates a typical transition of a conditional probability distribution of evoked responses, i.e., the number of evoked spikes recorded at the electrode before and after training. In this case, a typical shift of a peak of the (0,1) type (blue curve) is presented. Fig 4D shows the transition of responses over training at another stimulated electrode. In contrast to Fig 4C, a shift of a peak of the (1,0) type (red curve) is shown. The transition of response at each electrode can be found in S1 Dataset.

**Fig 5:**
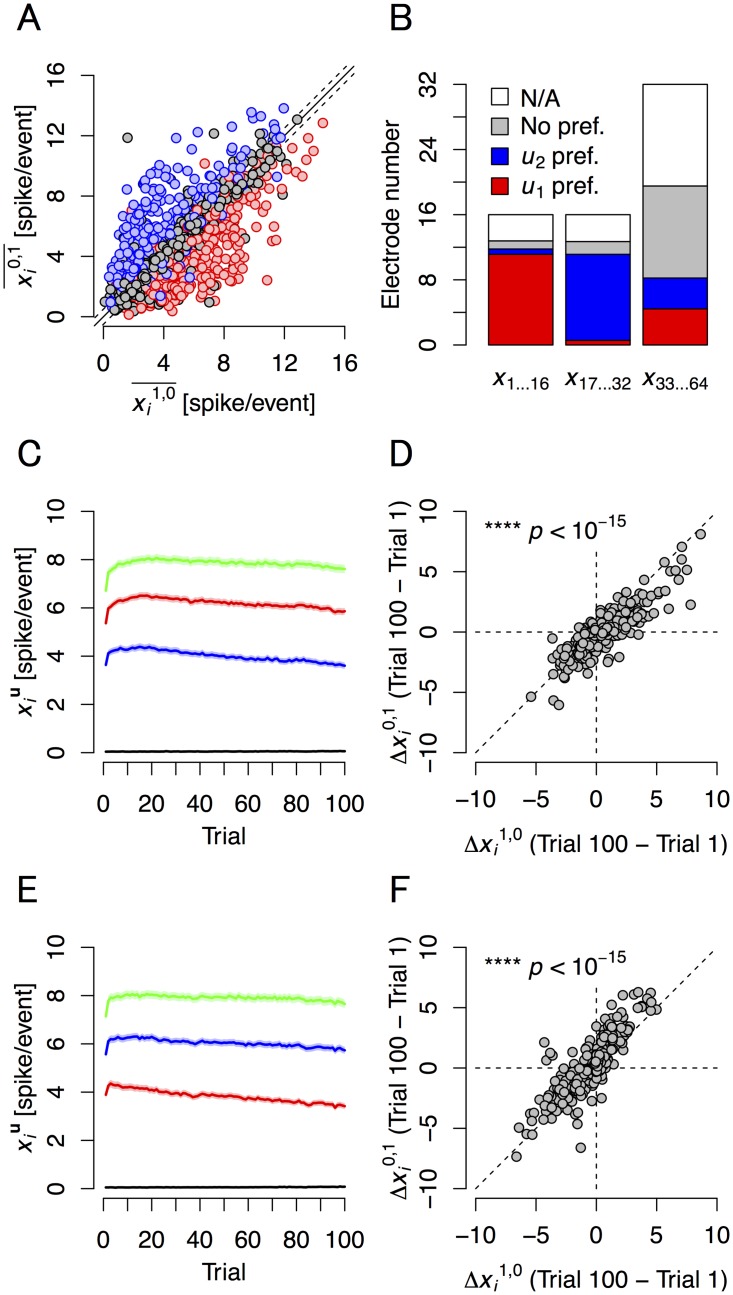
Response preference all-trial summary, transitions, and overall changes. **(A)** Response preference. Horizontal and vertical axes are the all-trial averages of the expectation of response when **u** = (1,0) and (0,1), respectively. Red circles are responses recorded with electrodes *x*_1_, …, *x*_16_, blue circles, with electrodes *x*_17_, …, *x*_32_, and black circles, with electrodes *x*_33_, …, *x*_64_. Circles are superimposed data from all cultures (*n* = 1035 electrodes from 23 cultures; the total number of electrodes was 1472, but 437 electrodes were not available, see (B)). The solid line is the identity line. Dashed lines indicate ± 0.5 spike/event. **(B)** The expectation of the numbers of four types of electrode responses in a culture. Red, blue, and gray correspond to the number of electrodes that record *u*_1_-preferring, *u*_2_-preferring, and neither-preferring responses, respectively. White shows the number of electrodes that were not available or suitable for analysis because of insufficient spikes. Neural activities were recorded from the majority of the electrodes. **(C)** Transitions of the expectation of response averaged over just the *u*_1_-preferring electrodes. As in Fig 4C, the four curve colors correspond to the four states of **u**. **(D)** The change in responses between trials 1 and 100. Horizontal and vertical axes plot the difference in the conditional expectations between trials 1 and 100 when **u** = (1,0) and (0,1), respectively. At *u*_1_-preferring electrodes, the increase of *x*_*i*_^1,0^ was significantly greater than that of *x*_*i*_^0,1^ (****, *p* < 10^−15^; *n* = 371 electrodes from 23 cultures). The dashed diagonal line is the identity line. **(E)** and **(F)** Same as (C) and (D), but averaged over just the *u*_2_-preferring electrodes. At *u*_2_-preferring electrodes, the increase of *x*_*i*_^0,1^ was significantly larger than that of *x*_*i*_^1,0^ (****, *p* < 10^−15^; *n* = 345 electrodes from 23 cultures). In (C) and (E), shadowed areas are S.E.M.

These results suggested that neurons near stimulated electrodes had preferences to one of the two hidden signals, but not the other. Specifically, most neurons from electrodes 1–16 (*x*_1_, …, *x*_16_) preferred *u*_1_ signals (neurons were activated more largely when **u** = (1,0) than when **u** = (0,1)), most neurons from electrodes 17–32 (*x*_17_, …, *x*_32_) preferred *u*_2_ signals, and most neurons at electrodes 33–64 (non-stimulated; *x*_33_, …, *x*_64_) showed no preference (Fig 5A and 5B). Note that *x*_*i*_^**u**^ indicates the conditional expectation with the source state **u** and $\bar{x_{i}{}^{u}}$ is its over-trial average. Neurons near stimulated electrodes exhibited larger responses compared to these near non-stimulated electrodes. In *u*_1_-preferring neurons, the increase in response strength was larger when the state of the source was **u** = (1,0) than when it was **u** = (0,1) (Fig 5C and 5D), while the exact opposite alteration profile was observed in *u*_2_-preferring neurons (Fig 5E and 5F). Moreover, at 50 electrodes out of 371 *u*_1_-preferring electrodes, $\bar{x_{i}{}^{1,0}}$ was 3 times larger than $\bar{x_{i}{}^{0,1}}$, and at 44 electrodes out of 345 *u*_2_-preferring electrodes, $\bar{x_{i}{}^{1,0}}$ was 3 times larger than $\bar{x_{i}{}^{1,0}}$ as all trial average (S1A Fig). Additionally, the number of such electrodes increased during training (S1B Fig). If a neuron responded to *s*_*i*_ (*i* = 1, …, 16), $\bar{x_{i}{}^{1,0}}$ should be 3 times as large as $\bar{x_{i}{}^{0,1}}$ by the relationship between *s*_*i*_ and **u**, while if a neuron responded to *s*_*i*_ (*i* = 17, …, 32), $\bar{x_{i}{}^{0,1}}$ should be 3 times as large as $\bar{x_{i}{}^{1,0}}$. Therefore, this indicates that at approximately 13% of *u*_1_- or *u*_2_-preferring electrodes, neural responses (*x*_*i*_) were more likely to be determined by the state of hidden sources (**u**) rather than by induced stimulation itself (*s*_*i*_) in the strict sense of the word. Taken together, these results suggest that neural responses were more likely determined by the state of hidden sources estimated based on inputs from multiple electrodes, termed source-coding, rather than the input from an electrode, e.g., the nearest electrode.

### Increased response specificity to discrete stimuli in cultured neuron networks

**Fig 6:**
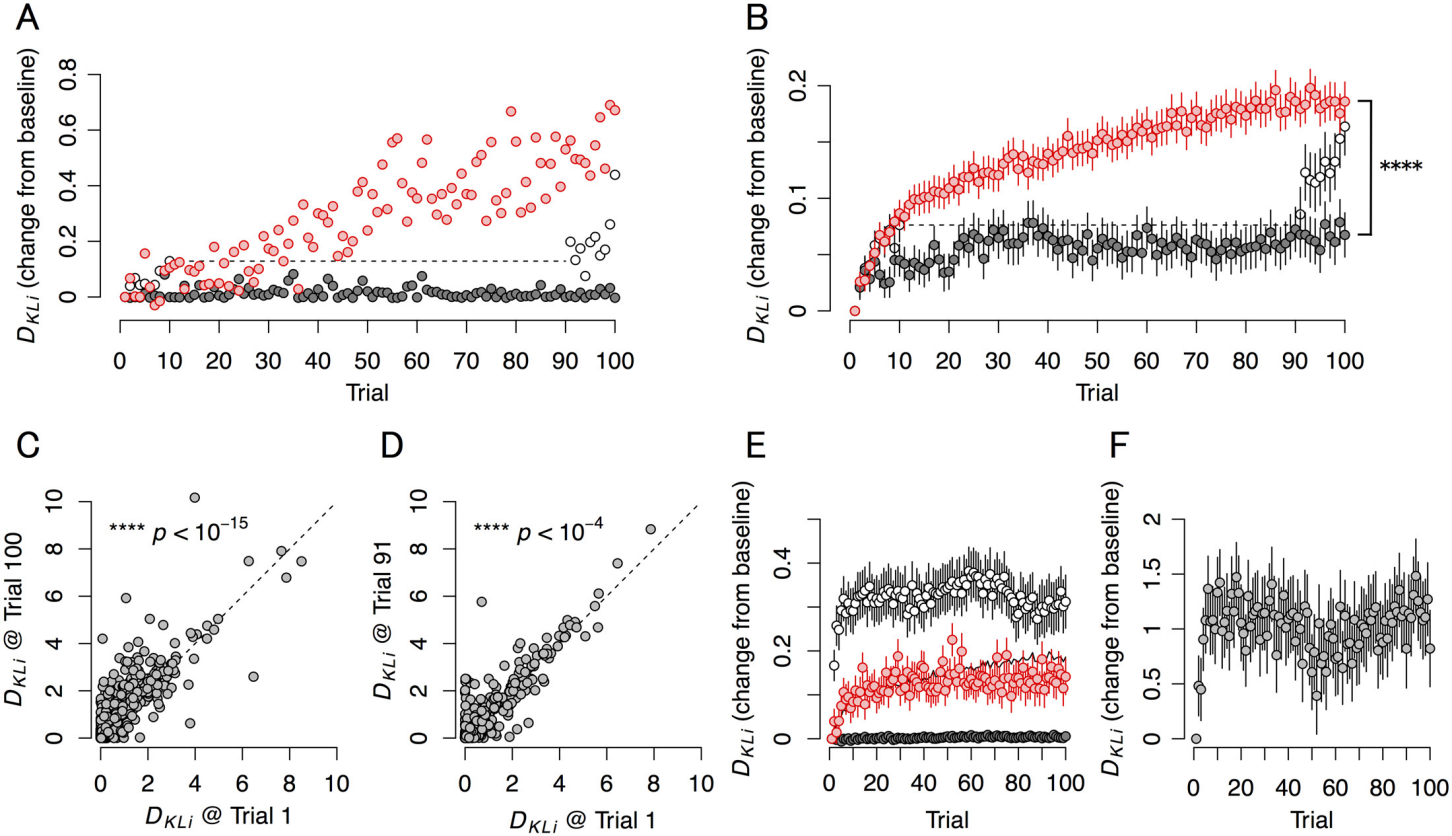
Alterations in Kullback-Leibler divergence (KLD) indicate distinct response sensitivity to different source stimuli. Panels show typical (A) and mean (B) KLD transition, overall change in full (C) and partial (D) training, and transitions with alternative parameter settings (E and F). **(A)** Typical transition of the KLD recorded with an electrode. Red circles are KLDs recorded in a trained culture (TRN). Black circles are KLDs recorded in a culture trained in the presence of APV. White circles are KLDs recorded in a partially trained culture (PRT), where the culture was trained for 10 trials, then not stimulated for 18–24 h, then trained for a further 10 trials. **(B)** The mean transition of the KLD. Red circles are the mean KLDs averaged over electrodes in the TRN group (*n* = 1035 electrodes from 23 cultures). Black circles are the mean KLDs in the presence of APV (*n* = 435 electrodes from 9 cultures). White circles are the mean KLDs in the PRT group (*n* = 473 electrodes from 10 cultures). At trial 100, KLDs in TRN were significantly larger than those in APV (****, *p* < 10^−5^). **(C)** The change of KLDs in TRN (trial 1 vs. trial 100). Circles are KLDs for each electrode. The dashed line is the identity line. KLDs significantly increased after training (****, *p* < 10^−15^). **(D)** The change in the KLDs in PRT (trial 1 vs. trial 91). KLDs at trial 91 were significantly larger than those at trial 1 (****, *p* < 10^−4^), indicating that the increase in KLD was maintained over the resting time. **(E)** The mean transition of the KLD with alternative parameter settings. Red, white, and black circles are KLDs with (merged balance (*a*), source firing probability (*ρ*)) = (3/4, 1/4), (3/4, 3/4), and (1/2, 1/2), respectively. The black curve is the mean KLD in the TRN group. **(F)** The mean transition of the KLD. Circles are KLDs with (*a*, *ρ*) = (1, 1/2). In (B), (E), and (F), bars are S.E.M.

The difference between the probability distribution at **u** = (1,0) and (0,1) is a well-established criterion to evaluate response preference, which in information theory is often defined by the Kullback-Leibler divergence (KLD) [11]. We calculated KLD of the evoked response at each electrode under the assumption that these conditional probabilities conformed to a Poisson distribution. We observed a significant change in KLD (represented as *D*_*KLi*_, where *i* = 1, ……, 64 is the index of electrodes) between distributions given the (1,0) state and (0,1) state (*P*(*x*_*i*_(*t*)| **u** = (1,0)) and *P*(*x*_*i*_(*t*)| **u** = (0,1), respectively). The values of *D*_*KLi*_ were increased in some electrodes after the training period (red circles in Fig 6A), where trained neuron cultures are labeled as TRN. Moreover, the mean values for *D*_*KLi*_ averaged across all recording electrodes increased after training (Fig 6B and 6C). The increase in the value of *D*_*KLi*_ in trained neuron cultures in the presence of 20 μM 2-Amino-5-phosphonopentanoic acid (APV), an N-methyl-D-aspartic acid (NMDA)-receptor inhibitor, was significantly smaller than in nontreated TRN cultures (black circles in Fig 6B; ****, *p* < 10^−5^). We confirmed that the alterations in KLD were maintained for a long time by comparing continuously stimulated trained neurons to partially trained (PRT) neurons. PRT neurons were trained for only 10 trials, then went unstimulated for 18–24 h (i.e. the resting period), and then went through 10 additional training trials. In PRT cultures, the values of *D*_*KLi*_ at trial 91 (i.e., first trial after the resting period) were significantly larger than that at trial 1 (Fig 6D); however, the difference was significantly smaller than the difference in *D*_*KLi*_ observed between trial 1 and 91 in TRNs (white circles in Fig 6B; ****, *p* < 10^−4^). Interestingly, the values of *D*_*KLi*_ at trial 100 in PRTs were almost same level as that at trial 100 in TRNs (*p* = 0.268). The transition of KLD at each electrode can be found in S1 Dataset.

KLD was affected by the merged balance of inputs (*a*) and the frequency of inputs (*ρ*). Specifically, we varied input balance by comparing the change of the *a*:1–*a* = 3/4:1/4 balance condition with that of the 0:1 and 1/2:1/2 balance conditions and the source condition with a *ρ* = 1/2 probability with a 1/4 and 3/4 probability (Fig 6E and 6F). Compared to the initial values (trial 1 vs. trial 100), KLD was not altered by inputs with 1/2:1/2 ratio of merged balance ((*a*, *ρ*) = (1/2, 1/2)) (black circles in Fig 6E; *p* = 0.515; *n* = 147 from 4 cultures), suggesting that input variance was necessary to elicit these changes. As both PCA and ICA rely on variations of input, these results are consistent with the hypothesis that cultured neural networks use ICA-like signal processing. When sources summed to one with a probability of 1/4, i.e., (*a*, *ρ*) = (3/4, 1/4) (red circles in Fig 6E), KLD increased after training (***, *p* < 10^−3^; *n* = 139 from 4 cultures; trial 1 vs. trial 100). Similarly, when (*a*, *ρ*) = (3/4, 3/4) (white circles in Fig 6E), KLD increased after training (****, *p* < 10^−7^; *n* = 234 from 6 cultures; trial 1 vs. trial 100). The change in KLD with (*a*, *ρ*) = (3/4, 1/4) was slightly smaller than when (*a*, *ρ*) = (3/4, 1/2) (*p* = 0.469, at trial 100), while the change in KLD with (*a*, *ρ*) = (3/4, 3/4) was slightly larger than when (*a*, *ρ*) = (3/4, 1/2) (*p* = 0.166, at trial 100). When the input balance was 1:0 (not merged; (*a*, *ρ*) = (1, 1/2)), a large increase of KLD was observed (Fig 6F; ****, *p* < 10^−4^; *n* = 161 from 4 cultures; trial 1 vs. trial 100), which is an analog of conventional pattern separation [34]. Note that to calculate Fig 6F, when the change in KLD form trial 1 was larger than 10 or smaller than –10, it was shifted to 10 or –10, respectively.

### A recognition model used by cultured neural networks

**Fig 7:**
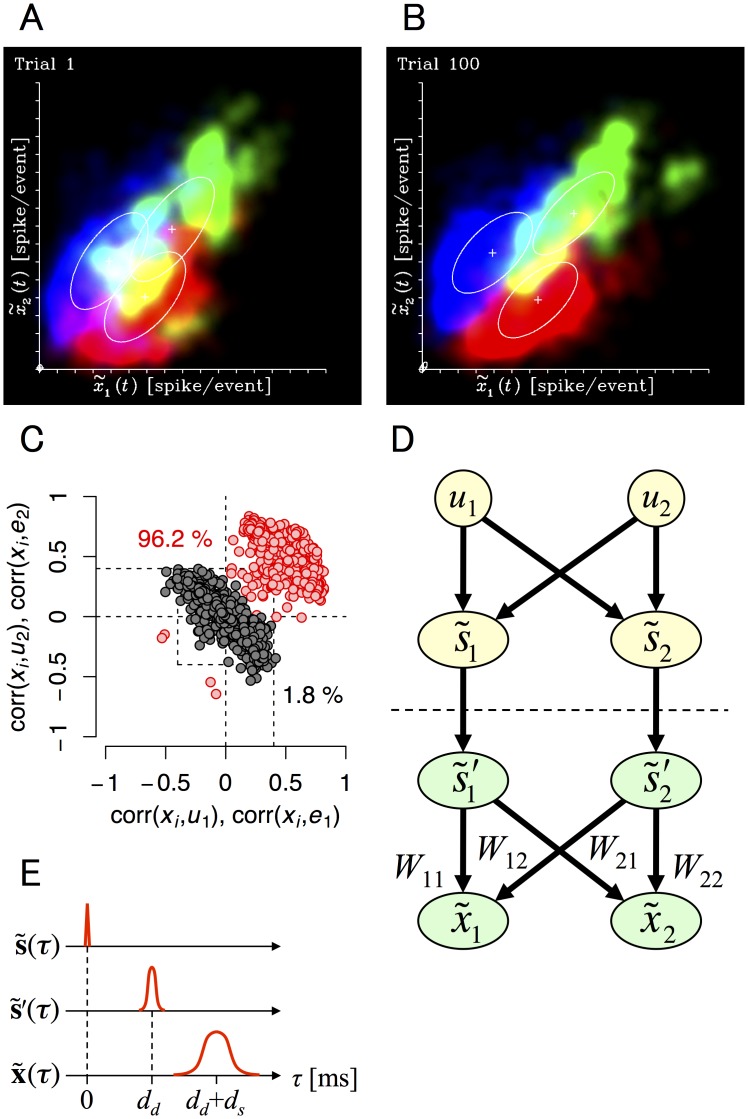
Population activity of cultured neural networks. Absence of stimulus classification before learning (A) and its presence after (B); summary of classification statistics (C); summary of the inverse recognition model (D) and dynamics of the inverse recognition model (E). **(A)** Evoked responses of populations of cultured neurons before training. Horizontal and vertical axes are the averaged responses of *u*_1_- and *u*_2_-preferring electrodes (${\tilde{x}}_{1}$ and ${\tilde{x}}_{2}$). Red, blue, and green indicate evoked responses when the state of **u** is (1,0), (0,1), and (1,1), respectively. The Fig corresponds to a superimposition of responses at *t* = 1, …, 256 from 23 cultures. Plus-marks and ellipses are the means and standard deviations of $\tilde{x}$ given **u** averaged over 23 cultures. The scale indicates the averaged spike number per stimulation. **(B)** Evoked responses after training. The evoked response transient throughout training is shown in S3 Movie. **(C)** The distribution of correlation. Red circles plot the correlation of *x*_*i*_ with *u*_1_ (horizontal) and *u*_2_ (vertical) (*n* = 1035 electrodes from 23 cultures). Black circles plot the correlation of *x*_*i*_ with *e*_1_ (horizontal) and *e*_2_ (vertical), where *e*_1_ and *e*_2_ are the error of *x*_*i*_ from *u*_1_ and *u*_2_, respectively. **(D)** Schematic image of a population model under the assumptions of the inverse recognition model. Our model assumes that $\tilde{s}$ is a column vector of inputs, $\tilde{s}$ **’** and $\tilde{x}$ are column vectors of the population activity of neuron groups, and *W* is a 2 × 2 matrix of connection strengths. Also, we assume that $\tilde{x}$ can be represented as a multiplication of the connection strength matrix *W* by $\tilde{s}$ **’** (linear, firing-rate neuron model). Based on the recognition model, *W* was calculated from the relationship between the amplitude of the stimulation and the evoked response using the maximum likelihood estimation. **(E)** Dynamics of the inverse recognition model. Upper, middle, and lower time courses represent input, direct response, and synaptic response, respectively.

We then set out to build a population-based model of neural network assembly based on our experimental paradigm. We defined the population model as $\tilde{x}={\left({\tilde{x}}_{1},{\tilde{x}}_{2}\right)}^{T}$, where ${\tilde{x}}_{1}$ and ${\tilde{x}}_{2}$ represent mean evoked responses of neurons in *u*_1_- and *u*_2_-preferring neuron groups in each culture preparation. Distribution of $\tilde{x}(t)$ at trial 1 and 100 are shown in Fig 7A and 7B, which represents the recognition density [19, 20] of $\tilde{x}$, $q(\tilde{x})$. Alterations observed in $q(\tilde{x})$ over the trial periods are show in S3 Movie. Notably, the total evoked response from all available electrodes (${\tilde{x}}_{1}+{\tilde{x}}_{2}$) was almost proportional to the total input (i.e., the number of stimulated electrodes) (S2A Fig).

Early computational studies proposed several learning models (recognition models) employing blind source separation. These models can be roughly separated into two types: the inverse recognition model [12–14, 44] and the feed-forward recognition model [15–17, 19, 20]. Considering the fact that inputs **s** were instantaneously induced in cultured neural networks and evoked responses recorded at stimulated electrodes decreased 20–30 ms after each stimulation (Fig 4A and 4B), the feed-forward recognition model was not suitable in this situation, as it requires the dynamics of neural networks to converge towards an equilibrium state for learning. Moreover, large populations of neurons that we observed were state-coding and correlated with sources (**u**) (96.2% of electrodes were corr(*x*_*i*_, *u*_1_) > 0.4 or corr(*x*_*i*_, *u*_2_) > 0.4), while only a small population of neurons were correlated with estimation errors (*e*_1_ or *e*_2_, where *e*_1_ and *e*_2_ are estimation errors of *x*_*i*_ from *u*_1_ and *u*_2_; only 1.8% of neurons were |corr(*x*_*i*_, *e*_1_)| > 0.4 or |corr(*x*_*i*_, *e*_2_)| > 0.4) (Fig 7C). Therefore, our results indicated that the recognition model used by cultured neural networks is more consistent with the inverse model, as the inverse model does not require the equilibrium state of $\tilde{x}$ or the existence of error-coding neurons. Based on this evidence, we generated an inverse recognition model of cultured neural networks, as we show in Fig 7D. Schematic images of the model’s dynamics are shown in Fig 7E. Taken together our results indicated that cultured neural networks implement ICA-like learning and that their dynamics can be described by an inverse recognition model.

### Connection strengths are altered according to the principle of free energy minimization

**Fig 8:**
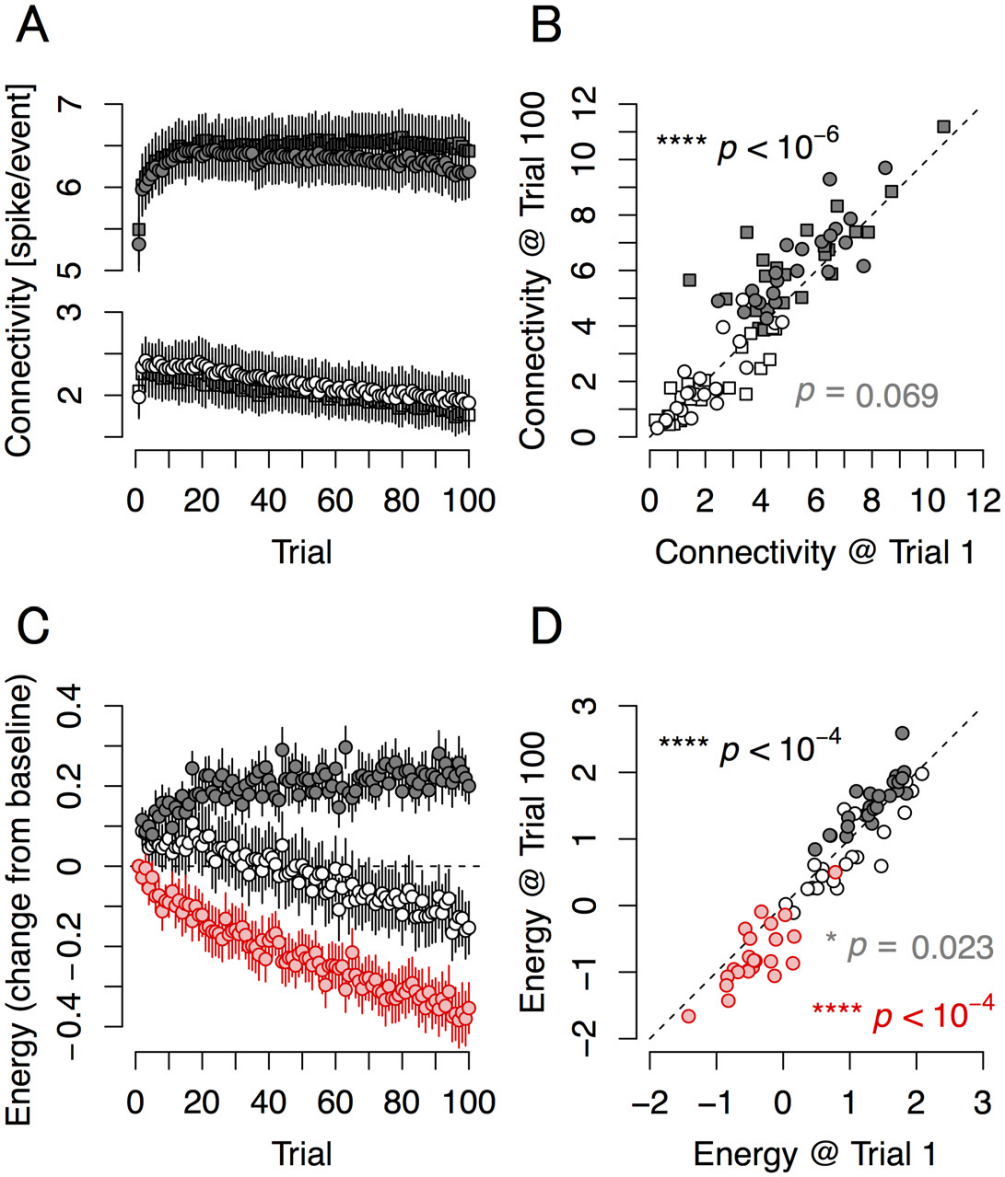
Free energy properties in cultured neural networks. Transitions during learning (left) and overall before-and-after changes (right) in connection strengths (top) and in three descriptors of evoked responses modeled on information-theoretic functions (bottom). **(A)** Connection strengths of the neural population estimated from the number of evoked response per trial. Black circles and squares are *W*_11_ and *W*_22_. White circles and squares are *W*_12_ and *W*_21_. Bars are S.E.M. **(B)** The change in connection strengths (trial 1 vs. trial 100). *W*_11_ and *W*_22_ significantly increased after training (****, *p* < 10^−6^; *n* = 46 from 23 cultures), while *W*_12_ and *W*_21_ tended to decrease (*p* = 0.069; *n* = 46 from 23 cultures). **(C)** Transition of the expectation of internal energy (〈*U*〉; white circles), Shannon entropy (*H*; black circles), and free energy (*F*; red circles) in cultured neural networks estimated from evoked responses per trial. Bars are S.E.M. **(D)** The change in 〈*U*〉, *H*, and *F* (trial 1 vs. trial 100). After training, the expectation of internal energy decreased (*, *p* = 0.023; *n* = 23 cultures), Shannon entropy significantly increased (****, *p* < 10^−4^; *n* = 23 cultures), and free energy significantly decreased (****, *p* < 10^−4^; *n* = 23 cultures). These results suggest that learning in cultured neural networks was governed by the free-energy principle.

Estimations of effective connectivity help in understanding neural dynamics [45, 46]. To estimate parameters of the inverse model from observed evoked responses, we calculated the maximum likelihood estimator of connectivity *W* (a 2×2 matrix) to analyze the averaged synaptic connection strengths within and between assemblies. Changes in estimated connection strengths are shown in Fig 8A. After training (relative to trial 1), intrinsic connection strengths (*W*_11_, *W*_22_) increased significantly, while connectivity between different neuron groups (*W*_12_, *W*_21_) tended to decrease (Fig 8B). Notably, if we assumed a constraint on total synaptic strengths with a *γ*-norm (the 1/*γ* power of the *γ* power sum of synaptic strengths), and if *γ* was between 2 and 4, the *γ*-norm of the connection strengths maintained almost same value during the latter part of the training period (S2B Fig).

As the model and connection parameters are well defined, we could calculate the internal energy and the Shannon entropy for these neural networks. To do this, we assumed that $q(\tilde{x})$ obeys a Gaussian mixture model with four peaks corresponding to the four states of **u**. Internal energy, $U\;=\;U\left(\tilde{s},\;\tilde{x},\;W\right)$, is defined as the negative log likelihood function of prediction error at a moment, where $\tilde{\text{s}}$ and $\tilde{x}$ are input and output, respectively. Shannon entropy, *H*, is defined by $H\;=\;H[q(\tilde{x})]$. Friston’s free energy, *F*, is defined as the difference between 〈*U*〉 and *H* [19, 20], where 〈•〉 is an expectation under $q(\tilde{x})$. Therefore, *F* is represented as $F(\tilde{s},\;\tilde{x},\;W)\;=\;\langle U(\tilde{s},\;\tilde{x},\;W)\rangle \;-\;H[q(\tilde{x})]$. Generally, free energy gives an upper bound on ‘surprise’ of inputs, so the decrease of free energy implies that the system is changing to adapt to (or learn) its environment [19, 20]. The full details of these calculations are fully described in the Methods. These components of free energy changed dramatically over training trials (Fig 8C). We found that the expectation of internal energy 〈*U*〉 decreased, Shannon entropy *H* increased significantly, and free energy *F* decreased significantly after training (Fig 8D), which is consistent with the principle of free-energy minimization [19, 20]. These data thus indicate that connectivities in neural networks were established such that they minimize free energy (*F*).

As expected, as learning proceeds over trials, the implicit entropy of the probabilistic encoding increases in accord with Jaynes’ maximum entropy principle [41, 42]. Crucially, this is accompanied by a profound decrease in energy (i.e., the amount of prediction error). Therefore, the decrease in the energy and the increase in the entropy both contributed to produce an overall reduction in free energy––that can only be attributed to learning or plasticity. This assertion was verified empirically by quantifying free energy changes in the presence of APV. Remarkably, free energy did not change at all during training under APV (S3 Fig).

### Learning rule of cultured neural networks

**Fig 9:**
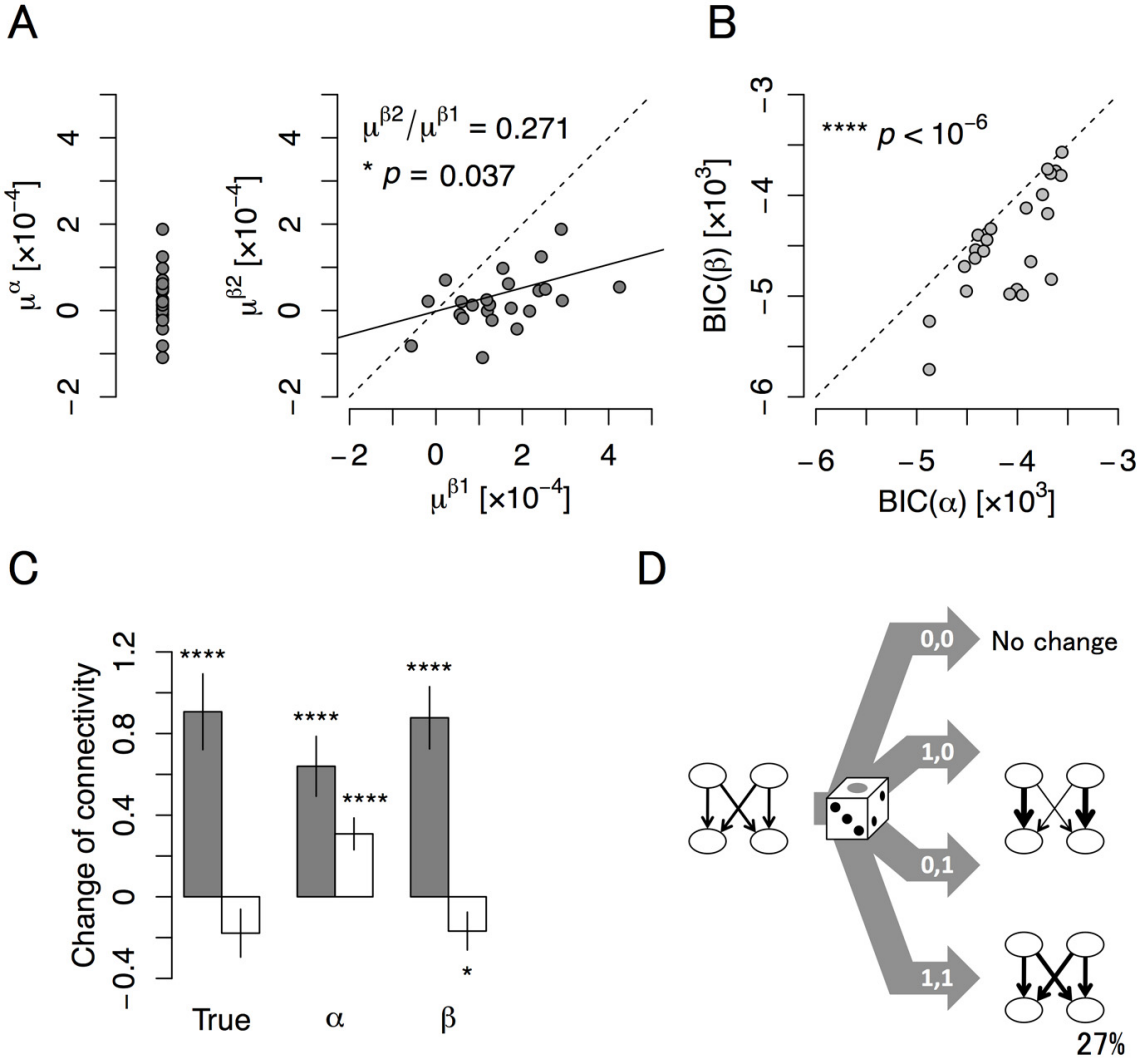
The governing learning rule in cultured neural networks. The rule was determined from (A) learning efficacies, (B) the Bayesian information criterion (BIC), and (C) model predictions of connectivity changes, and summarized in (D). **(A)** The expectation of learning efficacy estimated from connection strengths under the assumption that synaptic plasticity in cultured neural networks obeys either a Hebbian (*μ* ^*α*^; left) or state-dependent Hebbian (*μ* ^*β*1^, *μ* ^*β*2^; right) rule. *μ* ^*β*2^ was correlated with *μ* ^*β*1^ (*, *p* = 0.037; *n* = 23 cultures, Spearman test) and their ratio was 27.1%. **(B)** The BIC of the *α*- and *β*-models. BIC of the *β*-model was significantly smaller than that of the *α*-model (****, *p* < 10^−6^; *n* = 23 cultures). **(C)** Measured changes in connection strengths and those estimated from Hebbian and state-dependent Hebbian plasticity rules. Black bars are the mean ± S.E.M of the change (trial 100 –trial 1) in *W*_11_ and *W*_22_ (*n* = 46 from 23 cultures), which increased in all cases (****, *p* < 10^−5^). White bars are the mean ± S.E.M of the change in *W*_12_ and *W*_21_ (*n* = 46 from 23 cultures). *W*_12_ and *W*_21_ tended to decrease after training when calculated from response data (left; *p* = 0.069). *W*_12_ and *W*_21_ estimated from the *α*-model significantly increased (center; ****, *p* < 10^−4^). *W*_12_ and *W*_21_ estimated from the *β*-model significantly decreased (right; *, *p* = 0.023), in agreement with the experimentally determined results. **(D)** Suggested learning rule, which is based on state-dependent Hebbian plasticity, and leads to blind source separation. Synaptic plasticity in cultured neural networks almost followed a strict Hebbian rule. However, experimental data indicate that learning efficacy was not the same for each input state. Substantial Hebbian plasticity occurred when **u** = (1,0) or **u** = (0,1), while limited or anti-Hebbian plasticity occurred when **u** = (1,1). From the estimated efficacies *μ* ^*β*1^ and *μ* ^*β*2^, the efficacy of **u** = (1,1) was apparently only 27% of that of **u** = (1,0) or (0,1).

The changes in KLD and free energy we observed are indicative of synaptic plasticity and suggested that cultured neural networks are capable of performing blind source separation. These findings further suggested the existence of a transformation matrix (*W*) in cultured neural networks, which transforms merged inputs to independent outputs [12–14, 44]. However, it is unclear whether the blind source separation is realized only by Hebbian learning [18]. To estimate the learning rule of cultured neural networks, we first considered a simple Hebbian plasticity model, where a learning efficacy *α*_**u**_ becomes 0 for **u** = (0,0) and *α* for other states (*α*-model; see also the Methods). We then estimated *α* for each culture sample. The estimated values of *α* are shown in Fig 9A left and the Bayesian information criterion (BIC) [47] in *α*-model is shown in Fig 9B. In this *α*-model, connections between different neuron groups (*W*_12_, *W*_21_) were expected to increase substantially, because Hebbian learning operates by simply increasing the correlation among neurons that fire together (Fig 9C). However, we did not observe substantial increases between neuron groups, indicating that a simple Hebbian rule could not explain our experimental results.

These results therefore suggested that blind source separation in our cultured neural networks required another mechanism. We thus considered a modified version of Hebbian plasticity (*β*-model), where a learning efficacy *β*_**u**_ depends on the state of **u**, 0 for **u** = (0,0), *β*_1_ for **u** = (1,0), (0,1), and *β*_2_ for **u** = (1,1). *β*_1_ and *β*_2_ were estimated for each culture. Interestingly, we found that estimated values of *β*_2_ were significantly smaller than the estimated values of *β*_1_ (approximately 27% of *β*_1_; Fig 9A right). Moreover, the BIC was significantly smaller than in the *α*-model (Fig 9B). Accordingly, the *β*-model successfully explained the increase of intrinsic connections within neuron groups (*W*_11_, *W*_22_), and the absence of increases inter-connections between different groups (*W*_12_, *W*_21_) (Fig 9C). Furthermore, as an additional Bayesian model comparison, we showed that Hebbian plasticity with state-dependent efficacy (the *β*-model) is better than Hebbian plasticity with *γ*-norm constraint on total synaptic strength (the *α’*-model) to explain our experimental results (see S1 Note and S4 Fig).

These results suggest that cultured neural networks do not use the simplest form of the Hebbian plasticity rule (the *α*-model), but rather a state-dependent Hebbian plasticity rule (the *β*-model) in which learning efficacy is modified according to the state of sources. A conceptual conclusion is that the depression in inter-connections between different groups and the formation of cell assemblies are crucial to achieve blind source separation. Generally, the potentiation in connections makes the correlation between a neuronal group and a source stronger, while their depression makes the correlation between the neuronal group and the other source weaker. In our analysis, because the *β*-model encouraged stronger depression in connections from the other source and induced stronger competition between different neuronal groups, the *β*-model was better able to explain the results than the *α*-model. Moreover, this result supports the hypothesis that neurons render their activity independent of each other. This is consistent with early work on decorrelating or lateral interactions in PCA/ICA learning rules, which, importantly, can be formulated as variational free energy minimization [48].

## Discussion

In this study, we discovered that cultured neural networks were able to identify and separate two hidden sources. We found that the distinct classes of neurons learned to respond to the distinct hidden sources and that this was reflected in differences in the Kullback-Leibler divergence (KLD). We then sought to determine how connection strength is determined between cultured neurons and found that connectivities are established such that they minimize free energy. Finally, we integrated these data to construct a model of learning in cultured neural networks and determined that learning is established by a modified Hebbian plasticity rule. Taken together these data indicate that cultured neural networks can infer multivariate hidden signals through blind source separation.

Although cultured neural networks are random and may not have functional structures for signal processing before training, our data indicated that the process of training enables them to self-organize and obtain functional structures to separate two hidden signals though activity-dependent synaptic plasticity, such as spike-timing dependent plasticity (STDP) [49–51]. This process was a clear example of unsupervised learning [9–11] in cultured neural networks.

Previous studies have reported that the response electrode almost agrees with the stimulating electrode [43] and that the increase in response strength at stimulated electrode is larger than in the non-stimulated electrodes [52]; our results are consistent with these findings. Synaptic plasticity and inputs with different merged points of balance are necessary for learning to occur. As spikes observed less than 10 ms after stimulation in our culture system corresponded to responses directly evoked by electrical stimulation and artifacts (switching noise), we only assessed spikes more than 10 ms after stimulation. This allowed the analysis of changes in neural activity related to mechanisms of synaptic plasticity. Indeed, we observed that changes in KLD were inhibited by APV, strongly suggesting that learning mechanism was mediated by long-term synaptic plasticity regulated by NMDA-receptor signaling. As a further indication of a role for long-term synaptic plasticity, assays of partially stimulated cultures indicated the changes brought about by neural activation were maintained after 18–24 h without stimulation. Additionally, our results indicated that differences in the size of inputs were necessary for blind source separation in cultured neurons. Neurons with larger initial states of KLD tended to exhibit greater changes, suggesting that learning is nuanced by the initial input strengths as would be consistent with most forms of Hebbian learning [18].

Although the specificity of the neuronal response to hidden sources increases significantly, there remains a possibility that the neurons merely responded to their neighbor input stimulation. In fact, responding to neighbor stimulation might be enough to increase the response specificity in the current stimulation design. Indeed, in a large portion of electrodes, neural responses were affected by the input from an electrode. However, we found that at least at 13% of *u*_1_- or *u*_2_-preferring electrodes, neural responses were more likely to be determined by the state of hidden sources rather than by the input from an electrode, typically the nearest one, in the strict sense of the word. Moreover, the number of such electrodes increased during training. In short, this means we might be observing the superimposition of the response to the input from an electrode and the response corresponding to the state of hidden sources. Hence, to reduce the effect of neighbor stimulation site and emphasize the response determined by the state of hidden sources, we should search the optimal stimulation design for investigating blind source separation as future work.

Even in the presence of APV, KLD increased slightly. One explanation is that this is the result of an NMDA-R-independent form of learning. For example, it is known that synaptic plasticity independent of NMDA-R activity occurs at GABAergic synapses [53, 54], and could alter the neural network state to some degree. However, it could also be related to the drug’s imperfect blockade of NMDA-Rs.

In our experiments, evoked activities of cultured neurons were only synchronously generated immediately after each stimulation. This would be expected for both forward and inverse recognition models, given that the input was synchronous and instantaneous (discrete-time system), but the dynamics did not reach an equilibrium as is required for learning of a feed-forward model. Moreover, most neurons we observed were highly correlated with one of two sources (source-coding neurons). Taken together, these findings suggest that for our experimental protocol, the structure of cultured neural networks can be represented as a two-layer feed-forward network constructed from input and output layers and functioning as an inverse recognition model. However, it remains unclear which model applies to cultured neural networks with non-synchronous input.

Although some ICA models use information via non-local connections, several studies have proposed local rules that ICA can be constructed only using biologically plausible local connections [48, 55]. Internal energy, or negative log likelihood, also decreased after training, indicating that our culture neural networks also performed a maximum likelihood estimation or a maximum a posteriori estimation. Consequently, the free energy of the population model decreased significantly after training as predicted by the free energy principle [19, 20], which can also be regarded as an increase in mutual information between input and output (infomax principle) [56, 57]. Taken together these results suggest that in response to synchronous input, cultured neural networks perform ICA-like learning using an inverse recognition model constructed from local connections, and they adhere to the free-energy principle.

Experimental results suggest that the change in synaptic connection strengths in our model is better explained by a state-dependent Hebbian plasticity rule rather than the simplest Hebbian rule (Fig 9D). A possible explanation is as follows: Initially, many neurons may respond strongly to the nearest electrode but may also respond weakly to distant electrodes. According to Hebbian plasticity, synapses that respond to stimulation of the nearest electrode (and thus, to the source that tends to activate the nearest electrode) are likely potentiated because of the large postsynaptic response to the nearest electrode. If depression is induced in the other synapses in accordance with a plasticity rule, the neural response to the source that effectively stimulates the nearest electrode will be facilitated, while that to the other source will be depressed. This scenario seems to qualitatively explain the experimental results; however, our analysis implies that the simplest Hebbian plasticity (the *α*-model) cannot change synaptic strengths in this manner because of larger LTP in the **u** = (1,1) state than LTD in the **u** = (1,0) and (0,1) states, and that state-dependent Hebbian plasticity (the *β*-model) better explains the results since it suppresses LTP in the **u** = (1,1) state, providing stronger competition between neurons. Nevertheless, a more biologically plausible Hebbian plasticity model, such as the STDP model [21], should be analyzed in a systematic future study. It is unclear whether such a model fully explains the experimental results, and we would like to investigate this in the future.

Indeed, cultured neurons could not directly know the state of **u**; however, they could distinguish the state of **u** = (1,1) from other states as the total of evoked activity was significantly larger for this state than the other states. It is likely cultured neurons use the total of evoked activity to determine learning efficacy. Although we considered a 2-state model of learning efficacy, since the **u** = (1,0) and (0,1) states are symmetrical in our experimental setup, a 3-state model could be considered if presented with an asymmetrical stimulation pattern. Some mathematical models of ICA [44, 48, 55] conform to a modified form of Hebbian plasticity as well. Moreover, modulation of synaptic plasticity by GABAergic input [58, 59] may operate learning-efficacy modulation; nevertheless, additional experiments are necessary to determine the physiology of the modulation of Hebbian plasticity we observed.

It is known that animals have a high aptitude for pattern separation [60]. Spontaneous prior activity of a visual area learns the properties of natural pictures [28]. In the visual and olfactory systems, structures that decorrelate inputs and raise contrast are functional from birth [61, 62]. Although many studies of the pattern separation have been conducted, there is little research investigating blind source separation in biological neural circuits, as the decomposition of merged inputs is a more complex process than simple pattern separation. Owing to the simple properties of cultured neural networks, we observed the process by which neural networks actually learn to perform blind source separation. Evoked responses likewise changed after training to correspond to sources of the generative model. Practically, sensory inputs are a mixture of several sources except in a few ideal cases. Without blind source separation, these signals cannot be processed appropriately because the brain would fail to adequately decorrelate inputs. Therefore, our findings may be very important in understanding sensory perception.

Alternatively, one might consider that blind source separation can occur online, and seems to be more a matter of attention rather than learning, e.g., one can separate voices with different timbers at a cocktail party without experiences those particular timbers before. However, to direct one’s attention to a specific voice, the brain needs to separate a mixture of signals in advance. Therefore, although additional studies are required to explain the difference in the time scale of blind source separation between that considered in a cocktail party problem and that we observed, it is likely that ICA-like learning is necessary for blind source separation and a cocktail party effect.

Recently, the free-energy principle was proposed [19, 20], which specifically includes PCA/ICA, and has been applied to explain recognition models with highly hierarchical structure [17]. Cultured neural networks are useful to examine these theories as they can easily build any network structure [35, 36] and reproduce a variety of functions [30–39]. In addition, dynamic causal modeling for spike data [63] helps to investigate the detail structure of the recognition models of cultured neural networks. Moreover, there remains the possibility that cultured neural networks can perform even more complex types of unsupervised learning. These findings contribute not only to an increased understanding of learning and memory from a neuroscience perspective, but also in examining the free-energy principle at the cellular level.

In summary, we found that dissociated cultures of cortical neurons have the ability to carry out blind source separation in response to hidden signals. Learning in this paradigm used an inverse recognition model and was carried out according to a modified form Hebbian plasticity, which is likely regulated, at least in part, by NMDA signaling. These results are entirely consistent with the free-energy principle, suggesting that cultured neural networks perform blind source separation according to the free-energy principle. Most importantly, the free energy formulation allows us to quantify probabilistic encoding at the neuronal level in terms of information theory, and to test hypotheses about the changes in energy and entropy that are implicit in Bayes-optimal perception. We could have also assessed the accuracy and complexity of these representations with a slight change of variables. The free energy formalism prescribes Bayes-optimal update rules for the connection strengths that are associative in nature. Taken together these data provide a compelling framework for understanding the process by which the brain interprets hidden signals from complex multivariate information.

## Methods

### Cell cultures

All animal experiments were performed with the approval of the animal experiment ethics committee at the University of Tokyo (approval number, C-12-02, KA-14-2) and according to the University of Tokyo guidelines for the care and use of laboratory animals. The procedure for preparing dissociated cultured of cortical neurons was based on a modified version of the procedure described in a previous study [30]. Pregnant females of Wistar rat (Charles River Laboratories, Japan) were anaesthetized with isoflurane and immediately sacrificed. 19-day-old embryos (E19) were extracted and sacrificed by decapitation under ice-cold anesthesia. Cortical cells were removed from embryos and dissociated into single cells with Trypsin (Life Technologies) at 37°C for 20 min. The density of cells was adjusted to 1 × 10^7^ cells/mL. 5 × 10^5^ of the dissociated cells in 50 μL were seeded on the center of MEA dishes (Fig 1 and 1B), where the surface of MEA was previously coated with polyethyleneimine (Sigma-Aldrich) overnight. Note that to prepare high-density cultures, cells were dropped on the region where electrode terminals were disposed. The culture medium consisted of Dulbecco’s modified Eagle’s medium (DMEM) (Life Technologies) containing 10% heat-inactivated fetal bovine serum (FBS) (Cosmo Bio), 5% heat-inactivated horse serum (HS) (Life Technologies), and 5–40 U/ml penicillin/streptomycin (Life Technologies). After sitting undisturbed in the MEA dishes for 30 min, the fresh culture medium and medium conditioned for 3 days in glial cell cultures, were added into MEA dishes at a ratio of 1:1. The cells were cultivated in a CO_2_ incubator, an environment of 37°C and a 5% CO_2_/95% air concentration. Half of the culture medium was changed once every third day. These cultures were cultivated for 18 to 83 days before electrophysiological measurements. Although the electrophysiological properties of cultured cortical neurons change during development, it has been reported that at the stage of culture using our experiments, the spontaneous firing patterns of neurons have reached a developmentally stable period [64–66]. Note that same cultures were used more than once for experiments with other stimulation-pattern conditions since learning history with other stimulation-pattern did not affect our experiments and evaluations of results. We used 27 different cultures for 7 experiments, which were performed 40 ± 18 days after seeding.

### Recording

The MEA system (NF Corporation, Japan) was used for extracellular recording of cultured neural networks. Electrode terminals and circuits on MEA dishes were handmade using a photolithography technique. The 8×8 electrode terminals of MEA were disposed on a grid with 250-μm distance. Platinum black was coated on all 50-μm-each side electrode terminals. Neural signals were recorded with a 25 kHz sampling frequency and band-pass filtered between 500–2000 Hz, and were recorded over 14 h. All recordings and stimulation were conducted in a CO_2_ incubator. From the spike sorting analysis [67], an electrode was expected to record the activities from up to four neurons. For more details of MEA recording, see previous studies [30, 40].

### Electrical stimulation

Electrical stimulation was applied through 32 electrodes in pulse trains with 1 s intervals (Fig 2). Pulses were biphasic with each phase having a duration of 0.2 ms, and were delivered with 1 V amplitudes. Stimuli were delivered for each stimulating electrode only once in 1 s (1 Hz).

Before making inputs, we created hidden sources *u*_1_(*t*), *u*_2_(*t*), which corresponded to two independent random binary sources, *u*_1_(*t*), *u*_2_(*t*) ∈ {0,1} (*t* = 1, 2, …, 256 [s]). In this equation, *u*_1_(*t*) and *u*_2_(*t*) are signal patterns such that *u*_1_(*t*) = 0,1,0,0,0,1,1,0,1,0,1,0,0,1,1,0,… and *u*_2_(*t*) = 1,1,0,0,1,0,0,1,1,0,1,1,1,0,1,1,… as shown in Fig 2B and 2C. The value of *u*_1_(*t*) and *u*_2_(*t*) will be 1 with a probability of *ρ* = 1/2 at each time period. The terms *s*_1_(*t*), …, *s*_32_(*t*) correspond to merged inputs (electrical pulses), which were what we actually applied to cultured neurons. Therefore, cultured neurons did not know directly what the exact state of (*u*_1_(*t*), *u*_2_(*t*)) was because we did not induce (*u*_1_(*t*), *u*_2_(*t*)) directly.

The electrical stimulations (*s*_1_(*t*), …, *s*_32_(*t*)) were constructed from two independent binary sources, *u*_1_(*t*) and *u*_2_(*t*), in the following manner:

1. Values for half of the input train (*s*_1_(*t*), …, *s*_16_(*t*)) were randomly selected as *u*_1_(*t*) with a *a* = 3/4 probability, or *u*_2_(*t*) with a 1–*a* = 1/4 probability for each time period. This indicates, for example, that when *u*_1_(1) = 1 and *u*_2_(1) = 0, *s*_1_(1) = 1 would be expected to occur with 75% certainty and *s*_1_(1) = 0 would be expected to occur with 25% certainty. These expectations are common among *s*_1_(1), …, *s*_16_(1). As each component of *s*_1_(1), …, *s*_16_(1) was independently randomly selected, *s*_1_(1), …, *s*_16_(1) would become something like 1,0,1,1,0,1,1,1,1,1,1,1,0,1,0,1, which means, as population, 75% would be 1 and 25% would be 0 (although it is one example and the percentage would move stochastically). Thus, stimuli were chosen at random at each time period.
2. The values for the rest of trains (*s*_17_(*t*), …, *s*_32_(*t*)) were randomly selected by *u*_1_(*t*), with a 1–*a* = 1/4 probability, or *u*_2_(*t*) with that of *a* = 3/4. In other terms, the expectations of *s*_17_(*t*), …, *s*_32_(*t*) were exactly opposite to that of *s*_1_(*t*), …, *s*_16_(*t*).
3. The location of 32 stimulated electrodes corresponding to *s*_1_(*t*), …, *s*_32_(*t*) were randomly selected and fixed over trials. Stimulus evoked responses were recorded with the 64 MEA electrodes.

In other words, the generative model was composed of two hidden sources **u**(*t*) generated from the stationary Poisson process with the *ρ* intensity, **u**(*t*) ~ *Po*((*ρ*, *ρ*)^*T*^), 32 merged inputs **s**(*t*) generated from the non-stationary Poisson process with the time varying intensity of *A* **u**(*t*), **s**(*t*) ~ *Po*(*A* **u**(*t*)), and a 32 × 2 transform matrix *A*, in which (*A*_*i*1_, *A*_*i*2_) = (*a*, 1–*a*) for *i* = 1, …, 16 and (*A*_*i*1_, *A*_*i*2_) = (1–*a*, *a*) for *i* = 17, …, 32. Unless specifically mentioned, we used *ρ* = 1/2 and *a* = 3/4.

### Pharmacology

In the control condition, 2-Amino-5-phosphonopentanoic acid (APV) (a glutaminergic NMDA-receptor antagonist; Sigma-Aldrich) was used. APV was adjusted to 20 mM using PBS, and induced 2 μL into culture medium in an MEA dish to make a final concentration of 20 μM. After the injection, cultured neurons were placed for 30 min in a CO_2_ incubator, and stable activity of cultured neurons was confirmed before recording.

### Analysis

#### Spike detection

Before spike detection, artifacts were removed as follows: (i) values in saturated regions in raw data were detected and modified to 0, (ii) 500–2000 Hz band-pass filter were applied for the data, and (iii) values in regions that were modified in the first step were shifted to 0 again (see Fig 1C). Mean (*μ*) and standard deviation (*σ*) of extracellular potential (*v*) were calculated for each second. A spike was defined as the lowest point of a valley (*dv*/*dτ* = 0 and *dv* ^2^/*dτ* ^2^ > 0) that was lower than 5 times standard deviation (*v* − *μ* < −5 *σ*). Similar to previous study [67], if more than two spikes were detected during 0.25 ms, only a spike with lowest valley was chosen.

#### Conditional probability and expectation of a response

The Firing probability of neurons recorded at electrode *i* (*i* = 1, …, 64) is shown as *x*_*i*_(*τ*) [spike/ms]. The strength of evoked response against the *t*th stimulus (*x*_*i*_(*t*) [spike/event]; *t* = 1, …, 256) is defined as the number of spikes generated until 10–30 ms after each stimulation,

$$x_{i}\left(t\right)\;=\;\int_{1000t+10}^{1000t+30}x_{i}(\tau )d\tau .$$ (1)

Using histogram method, conditional probability distribution *P*(*x*_*i*_(*t*)| **u**(*t*) = **u**) is non-parametrically calculated (Fig 4C and 4D), where **u** = (*u*_1_, *u*_2_)^*T*^ is a column vector of the source state. Moreover, as the parametric method, we assume that the probability distribution of *x*_*i*_(*t*) given **u**(*t*) obeys the Poisson distribution, which is given by

$$P\left(x_{i}\left(t\right)|u\left(t\right)=u\right)\;=\;\frac{{\left(x_{i}{}^{u}\right)}^{x_{i}(t)}\text{exp}(-x_{i}{}^{u})}{(x_{i}(t))!},$$ (2)

where *x*_*i*_^**u**^ (a parameter of Poisson distribution) was a conditional expectation of *x*_*i*_(*t*) when the state of **u** is given. The maximum likelihood estimator of *x*_*i*_^**u**^ was defined as *x*_*i*_^**u**^ = *E*[*x*_*i*_(*t*)| **u**(*t*) = **u**, *t* = 1,…,256], where *E*[•] indicates the expectation, i.e., *x*_*i*_^**u**^ is a mean value of *x*_*i*_(*t*) when **u**(*t*) = **u**. *x*_*i*_^**u**^ was calculated for each trial. All trial average of *x*_*i*_^**u**^ is represented as $\bar{x_{i}{}^{u}}$. We only evaluated electrode with $(\bar{x_{i}{}^{0,0}}+\bar{x_{i}{}^{1,0}}+\bar{x_{i}{}^{0,1}}+\bar{x_{i}{}^{1,1}})/4\;\geq \;1$ spike/event as a recording electrode to be used for analysis. We assumed that a neuron group recorded at electrode *i* was *u*_1_-preferring when $\bar{x_{i}{}^{1,0}}\;-\;\bar{x_{i}{}^{0,1}}\;\geq \;0.5$ spike/event, *u*_2_-preferring when $\bar{x_{i}{}^{1,0}}\;-\;\bar{x_{i}{}^{0,1}}\;\leq \;-0.5$ spike/event, and no preference when otherwise. These neurons were categorized into *G*_1_ (*u*_1_-preferring), *G*_2_ (*u*_2_-preferring), and *G*_0_ (no preference), respectively.

#### Kullback-Leibler divergence

The Kullback-Leibler divergence (KLD) is the distance of two probability distributions [11]. KLD between *P*(*x*_*i*_(*t*)| **u**(*t*) = (1,0)) and *P*(*x*_*i*_(*t*)| **u**(*t*) = (0,1)) was defined by

$$\begin{array}{l} D_{KLi}\;=\;D_{KL}\left[P\left(x_{i}\left(t\right)|u\left(t\right)=\left(1,0\right)\right)|\left|P(x_{i}\left(t\right)\right|u\left(t\right)=\left(0,1\right)\right)]\\ =\;\sum_{m=0}^{\infty }\text{log}\frac{P(x_{i}(t)=m|u(t)=(1,0))}{P(x_{i}(t)=m|u(t)=(0,1))}P(x_{i}(t)=m|u(t)=(1,0))\\ =\;{\langle \text{log}P\left(x_{i}\left(t\right)|u\left(t\right)=\left(1,0\right)\right)\;-\text{ log}P\left(x_{i}\left(t\right)|u\left(t\right)=\left(0,1\right)\right)\rangle }_{P}{}_{\left(xi\left(t\right)|u\left(t\right)=\left(1,0\right)\right)}, \end{array}$$ (3)

where 〈•〉_*P*(*xi*(t)| **u**(*t*) = (1,0))_ is an expectation around *P*(*x*_*i*_(*t*)| **u**(*t*) = (1,0)) (Malkov bracket). Since we assume that *P*(*x*_*i*_(*t*)| **u**(*t*) = (1,0)) and *P*(*x*_*i*_(*t*)| **u**(*t*) = (0,1)) obey Poisson distribution, Eq 3 was calculated as

$$D_{KLi}=\;\left(\text{log}x_{i}{}^{1,0}-\text{log}x_{i}{}^{0,1}\right)x_{i}{}^{1,0}-x_{i}{}^{1,0}+x_{i}{}^{0,1}.$$ (4)

KLD is a non-negative value and becomes 0 if and only if two probability distributions are exactly equal. When the difference between two distributions is small, KLD becomes a small value; when the difference is large KLD becomes a large value.

#### Statistical test

The Wilcoxon signed-rank test was used as a paired testing. The Mann-Whitney *U* test was used as an unpaired testing. The Spearman test was used as a test of no correlation.

#### Modeling

Neurons in a culture that respond to stimulation with the same property were assumed to be in the same cell assembly, such that we considered the population model constructed from groups of *u*_1_- and *u*_2_-preferring neurons. Thus, we defined $\tilde{x}(t)={\left({\tilde{x}}_{1}(t),{\tilde{x}}_{2}(t)\right)}^{T}$ [spike/event] by

$$\begin{array}{l} {\tilde{x}}_{1}\left(t\right)\;=\;E[x_{i}\left(t\right)|i\in G_{1}],\\ {\tilde{x}}_{2}\left(t\right)\;=\;E[x_{i}\left(t\right)|i\in G_{2}]. \end{array}$$ (5)

Furthermore, we assumed that the recognition model used by cultured neurons is the inverse model with linear firing function, which is represented as

$$\begin{array}{l} {\tilde{s}}^{\prime }(\tau )=\tilde{s}(\tau -d_{d}),\\ \tilde{x}(\tau )=W\;{\tilde{s}}^{\prime }(\tau -d_{s})\;+\;\xi (\tau ), \end{array}$$ (6)

where $\tilde{x}(\tau )$, $\tilde{s}’(\tau )$ [spike/ms] and $\tilde{s}(\tau )$ [event/ms] are column vectors of synaptic response, direct response, and input with continuous time (*τ* [ms]). **ξ**(*τ*) [spike/ms] is a background noise and were assumed to obey a Gaussian distribution **ξ**(*τ*) ~ *N*(**ξ**; **0**, *Σ* ^*ξ*^). *W* [spike/event] is a 2×2 connection strength matrix representing identical connections and connections between two groups (Fig 7D). Note that *d*_*d*_ [ms] and *d*_*s*_ [ms] were latencies of responses directly evoked by stimulations and indirectly evoked via synaptic connections. It is known that direct responses evoked by extracellular stimulation are highly reproducible with small time variance, while indirect responses via synaptic connection have larger time variance [43]. Therefore, although the direct response $\tilde{s}’(\tau )$ was difficult to observe, due to the artifact and saturation, evoked responses against pulse inputs could be regarded as a two-layer feed-forward model, which is the same form as a linear firing rate neuron model constructed from input and output layers [12–14, 44]. As input was induced at a moment (assuming *τ* = 0), using the discrete time *t*, the response around *τ* = *d*_*d*_ + *d*_*s*_ could be represented as

$$\tilde{x}(t)=W\;\tilde{s}(t)\;+\;\xi (t),$$ (7)

where $\tilde{s}(t)$, a column vector, is defined by $\tilde{s}(t)\;=\;{\left({\tilde{s}}_{1},{\tilde{s}}_{2}\right)}^{T}\;=\;{\left(E\left[s_{i}(t)|\;i\;=\;1,\;\ldots ,\;16\right],\;E\left[s_{i}(t)|\;i\;=\;17,\;\ldots ,\;32\right]\right)}^{T}$. A schematic image of the dynamics of the model is shown in Fig 7E.

Generally, inverse recognition models [12–14, 44] (e.g., **x** = *W*_*inv*_ **s**, where **s** and **x** are input and output vectors, and *W*_*inv*_ is a transform matrix corresponding to synaptic connection strengths) learn the inverse of a transformation matrix *A* (*W*_*inv*_ = *A* ^–1^), i.e., *W*_*inv*_ converges to *A* ^–1^ after learning, where *A* is a transform matrix of sources (**u**) to inputs (**s**) in the generative model, **s** = *A* **u**. Whereas, feed-forward recognition models [15–17] (e.g., an equilibrium state can be represented as *W*_*for*_ **x** = **s**) learn *A* itself, i.e., a connection strength matrix *W*_*for*_ converges to *A* after learning. Because the model we assumed was constructed from a two-layer feed-forward model and *W* was expected to converge to *A* ^–1^, our model is categorized into the inverse model.

#### Cross-correlation between each electrode and population

Cross-correlation between *x*_*i*_(*t*) and **u**(*t*) is defined by $\text{corr}\left(x_{i},\;u\right)\;=\;\left(\text{cov}\left(x_{i},\;u_{1}\right)/\sqrt{\text{Var}(x_{i})\text{Var}(u_{1})},\text{ cov}\left(x_{i},\;u_{2}\right)/\sqrt{\text{Var}(x_{i})\text{Var}(u_{2})}\right)$, where cov(*x*_*i*_, **u**) is covariance between *x*_*i*_(*t*) and **u**(*t*), and Var(*x*_*i*_) and Var(**u**) are variance of them. Then, error of $\tilde{x}$ from **u** is defined by $e_{i}(t)\;=\;{\tilde{x}}_{i}(t)\;-\;\left(\Sigma_{t=1}{}^{256}\;{\tilde{x}}_{i}(t)\right)/\left(\Sigma_{t=1}{}^{256}\;u_{i}(t)\right)\;u_{i}(t)$, *i* = 1, 2. We also defined cross-correlation between *x*_*i*_(*t*) and **e**(*t*) by $\text{corr}\left(x_{i},\;e\right)\;=\;\left(\text{cov}\left(x_{i},\;e_{1}\right)/\sqrt{\text{Var}(x_{i})\text{Var}(e_{1})},\text{ cov}\left(x_{i},\;e_{2}\right)/\sqrt{\text{Var}(x_{i})\text{Var}(e_{2})}\right)$. corr(*x*_*i*_, **u**) and corr(*x*_*i*_, **e**) were used for evaluating whether *x*_*i*_(*t*) was state-coding (representing *u*_1_, *u*_2_) or error-coding (representing *e*_1_, *e*_2_) (Fig 7C).

#### Estimation of connection strengths

Internal energy $U(\tilde{s},\;\tilde{x},\;W)$ is defined as a negative log likelihood function, $U(\tilde{s},\;\tilde{x},\;W)\;=\;-\text{log }p\left(\xi |\;W\right)$. Note that **ξ** is regarded as the difference between an actual output, $\tilde{x}$, and an expected output *W* $\tilde{s}$. Thus, **ξ** is the error for a kind of optimal decoder, and $U(\tilde{s},\;\tilde{x},\;W)$ indicates an amount of prediction error. Since we assumed **ξ** obeys Gaussian distribution, **ξ** ~ *N*(**ξ**; **0**, *Σ* ^*ξ*^), we get

$$\begin{array}{l} U(\tilde{s}\left(t\right),\tilde{x}\left(t\right),W)\;=\;\frac{1}{2}\xi^{T}(\Sigma^{\xi }){}^{-1}\xi +\frac{1}{2}\text{log}{\left(2\pi \right)}^{N}\left|\Sigma^{\xi }\right|\\ =\;\frac{1}{2}{\left(\tilde{x}(t)-W\;\tilde{s}(t)\right)}^{T}(\Sigma^{\xi }){}^{-1}\left(\tilde{x}(t)-W\;\tilde{s}(t)\right)+\frac{1}{2}\text{log}{\left(2\pi \right)}^{N}\left|\Sigma^{\xi }\right|. \end{array}$$ (8)

As there are no hidden states and hyper-parameters, the expectation of *W* can be estimated using the conventional maximum a posteriori estimation, which is analog of the conventional model-based connection strength estimation [63, 68]. Since we assumed *W* obeys a Gaussian distribution *W* ~ *q*(*W*) = *N*(*W*; *μ* ^*W*^, *∑* ^*W*^) and the change in *W* during a trial is small, the mean value of *W*, *μ* ^*W*^, is given by *W* that minimizes the internal action $\bar{U}\;=\;\Sigma_{t=1}{}^{256}\;U\left(\tilde{s}(t),\;\tilde{x}(t),\;W\right)$. By solving the extreme value of $\bar{U}$, $\partial \bar{U}/\partial W\;=\;0$, we obtain

$$\mu^{W}\;=\;\left(\sum_{t=1}^{256}\tilde{x}(t)\tilde{s}{\left(t\right)}^{T}\right){\left(\sum_{t=1}^{256}\tilde{s}(t)\tilde{s}{\left(t\right)}^{T}\right)}^{-1}.$$ (9)

*μ* ^*W*^ was calculated for each trial. Thereby, we obtained the model, states, and parameters for both the generative and recognition models.

#### Estimation of internal energy, Shannon entropy, and free energy for neurons

Next, we calculated the free energy for neurons according to the free-energy principle [19, 20]. As above, the internal energy for neurons was defined by $U(\tilde{s},\;\tilde{x},\;W)\;=\;-\text{log }p\left(\xi |\;W\right)$, which describes amount of prediction error. In the recognition model of neurons $q(\tilde{x},\;W)$, the posterior on the activity of state coding neurons $\tilde{x}$ and the posterior on parameters *W* can be regarded as independent, $q(\tilde{x},\;W)\;=\;q(\tilde{x})\;q(W)$. We have already obtained *q*(*W*) as a Gaussian distribution. On the other hand, as shown in Fig 7A and 7B, $q(\tilde{x})$ cannot be readily regarded as a Gaussian distribution. Thus, we assumed $q(\tilde{x})$ would be a Gaussian mixture model with 4 peaks corresponding to 4 stimulus source states. Specifically, $q(\tilde{x})$ is represented as

$$\begin{array}{l} q(\tilde{x})\;=\;\frac{1}{4}\sum_{m=1}^{4}N(\tilde{x};\;\mu^{xm},\;\Sigma^{xm})\\ =\;\frac{1}{4}\sum_{m=1}^{4}\text{exp}\left\{-\frac{1}{2}{\left(\tilde{x}-\mu^{xm}\right)}^{T}{\left(\Sigma^{xm}\right)}^{-1}\left(\tilde{x}-\mu^{xm}\right)-\frac{N}{2}\text{log}2\pi -\frac{1}{2}\text{log}\left|\Sigma^{xm}\right|\right\}, \end{array}$$ (10)

where $q\left(\tilde{x}|\;u=\left(0,0\right)\right)$, $q\left(\tilde{x}|\;u=\left(1,0\right)\right)$, $q\left(\tilde{x}|\;u=\left(0,1\right)\right)$, and $q\left(\tilde{x}|\;u=\left(1,1\right)\right)$ are represented as $N\left(\tilde{x};\;\mu^{x1},\;\Sigma^{x1}\right)$, $N\left(\tilde{x};\;\mu^{x2},\;\Sigma^{x2}\right)$, $N\left(\tilde{x};\;\mu^{x3},\;\Sigma^{x3}\right)$, and $N\left(\tilde{x};\;\mu^{x4},\;\Sigma^{x4}\right)$, respectively. Estimators of **μ** ^*xm*^s, *Σ* ^*xm*^s and *Σ* ^*ξ*^ are calculated as

$$\begin{array}{l} \mu^{xm}=E\left[\tilde{x}\left(t\right)|u\left(t\right)=u,\;t=\;1,\ldots ,256\right]\;=\;{\left(E\left[x_{i}{}^{u}|i\in G_{1}\right],\;E\left[x_{i}{}^{u}|i\in G_{2}\right]\right)}^{T},\\ \Sigma^{xm}=E\left[\left(\tilde{x}\left(t\right)\;–\;\mu^{xm}\right)\;{\left(\tilde{x}\left(t\right)\;–\;\mu^{xm}\right)}^{T}|u\left(t\right)=u,\;t=\;1,\ldots ,256\right],\\ \Sigma^{\xi }=E\left[\left(\tilde{x}\left(t\right)\;–\;\mu^{W}\tilde{s}\left(t\right)\right)\;{\left(\tilde{x}\left(t\right)\;–\;\mu^{W}\tilde{s}\left(t\right)\right)}^{T}|u\left(t\right)=u,t=\;1,\ldots ,256\right], \end{array}$$ (11)

where **u** becomes (0,0), (1,0), (0,1), and (1,1) when *m* is 1, 2, 3, and 4, respectively. The expectation of $U(\tilde{s},\;\tilde{x},\;W)$ is given by

$${\langle U(\tilde{s},\tilde{x},W)\rangle }_{q(\tilde{x},W)}\;=\;1\;+\text{log }2\pi +\frac{1}{2}\;\text{log}\left|\Sigma^{\xi }\right|.$$ (12)

Shannon entropy of $q(\tilde{x})$ is given by

$$H[q(\tilde{x})]\;=\;{\langle -\text{log}q(\tilde{x})\rangle }_{q(\tilde{x})},$$ (13)

which is approximated as $H[q(\tilde{x})]\;=\;-1/256\;\Sigma_{t=1}{}^{256}\text{ log }q(\tilde{x}(t))$. As Shannon entropy of *q*(*W*), *H*[*q*(*W*)], only depends on $\tilde{s}(t)$ and is a constant over trials, we omit *H*[*q*(*W*)]. Accordingly, the free energy for neurons $F(\tilde{s},\;\tilde{x},\;\mu^{W})$ is represented as

$$\begin{array}{l} F(\tilde{s},\tilde{x},\mu^{W})\;=\;{\langle U(\tilde{s},\tilde{x},W)\rangle }_{q(\tilde{x},W)}\;-\;H[q(\tilde{x})]\\ =\;\frac{1}{2}\text{log}\left|\Sigma^{\xi }\right|\;+\;\frac{1}{2}\sum_{t=1}^{256}\text{log}q(\tilde{x}\left(t\right))\;+\;const. \end{array}$$ (14)

$F(\tilde{s},\;\tilde{x},\;\mu^{W})$ is an upper bound of surprise of input and becomes minimum if and only if $q(\;\tilde{x},\;W)$ is the same as the generative model (the true distribution of source).

#### Estimation of learning efficacy

Learning of cultured neural networks is assumed to obey Hebbian plasticity [18], which is represented as

$$dW=\alpha_{u}\langle \left(\tilde{x}-\langle \tilde{x}\rangle \right)\;{\left(\tilde{s}-\langle \tilde{s}\rangle \right)}^{T}\rangle +\varepsilon^{\alpha },$$ (15)

where 〈•〉 is an expectation around $p(\tilde{x})$ and *α*_**u**_ is a learning efficacy depending on state of **u** (*α*_0,0_, *α*_1,0_, *α*_0,1_, and *α*_1,1_ are efficacies at the condition of **u** = (0,0), (1,0), (0,1), and (1,1), respectively). *ε* ^*α*^ is a 2×2 matrix that represents the error and its elements are assumed to be independent of each other. We defined Eq 15 as an *α*-model. Eq 15 can be derived from the additive STDP model [21] when the source state changes rapidly. The aim is to estimate the value of *α*_**u**_. Let us set *z*_*ij*_^**u**^ as $z_{ij}{}^{u}\;=\;\Sigma_{t\in \left\{t|u(t)=u\right\}}\;\left({\tilde{x}}_{i}(t)\;-\;\langle {\tilde{x}}_{i}\rangle \right)\;\left({\tilde{s}}_{i}(t)\;-\;\langle {\tilde{s}}_{i}\rangle \right)$, which is an element of a 2×2 matrix *z* ^**u**^. We assume *α*_0,0_ = 0 since without activation, activity-dependent synaptic plasticity does not occur. As a simple Hebbian rule, we also assumed *α*_10_ = *α*_01_ = *α*_11_ = *α*, i.e., learning efficacies were common for all states of **u** except **u** = (0,0). As Eq 15 is rewritten as *dW*_*ij*_ = *α* (*z*_*ij*_^1,0^ + *z*_*ij*_^0,1^ + *z*_*ij*_^1,1^) + *ε* ^*α*^, under the assumption that *p*(*ε* ^*α*^_*ij*_| *α*) is a Gaussian distribution *N*(*ε* ^*α*^_*ij*_; 0, *Σ* ^*εαij*^), the negative log likelihood function for *α* is defined by

$$\begin{array}{l} L_{\alpha }\;=\;-\sum_{i}{}_{,j}\text{log}N\left(\varepsilon^{\alpha }{}_{ij};\;0,\Sigma^{\varepsilon \alpha ij}\right)\\ =\;\frac{1}{2}\sum_{l=1}^{100}\sum_{i,j}^{}\frac{1}{2\Sigma^{\varepsilon \alpha ij}}{\left\{dW_{ij}\left(l\right)\;-\;\alpha \left(z_{ij}{}^{1,0}\left(l\right)\;+\;z_{ij}{}^{0,1}\left(l\right)\;+\;z_{ij}{}^{1,1}\left(l\right)\right)\right\}}^{2}\;+\;50\sum_{i,j}\text{log}2\pi \left|\Sigma^{\varepsilon \alpha ij}\right|, \end{array}$$ (16)

where *dW*_*ij*_(*l*) and *z*_*ij*_^**u**^(*l*) are the change of *W*_*ij*_ in *l*th trial and *z*_*ij*_^**u**^ in *l*th trial. Since *dW*_*ij*_ is noisy and saturated in latter part, we assume *dW*_*ij*_(*l*) = (*W*_*ij*_(100) − *W*_*ij*_(1))/100. Additionally, we assume *Σ* ^*εαij*^s are common among all *i* and *j*. From Eq 16, under the assumption that *α* obeys a Gaussian distribution *q*(*α*) = *N*(*α*; *μ* ^*α*^, *Σ* ^*α*^), the expectation of *α* that gives the minimum of *L*_*α*_ is given by

$$\mu^{\alpha }\;=\;\frac{\Sigma_{l=1}^{100}\Sigma_{i,j}^{}dW_{ij}(l)\left(z_{ij}{}^{1,0}(l)+z_{ij}{}^{0,1}(l)+z_{ij}{}^{1,1}(l)\right)}{\Sigma_{l=1}^{100}\Sigma_{i,j}^{}{\left(z_{ij}{}^{1,0}(l)+z_{ij}{}^{0,1}(l)+z_{ij}{}^{1,1}(l)\right)}^{2}}.$$ (17)

Next, we considered the situation where learning efficacies were different depending on the condition of **u** (*β*-model). We define a learning efficacy *β*_**u**_ (*β*_0,0_, *β*_1,0_, *β*_0,1_, and *β*_1,1_) as a function of **u**. We assumed *β*_0,0_ = 0, *β*_1,0_ = *β*_0,1_ = *β*_1_ since **u** = (1,0) and (0,1) are symmetric, and *β*_1,1_ = *β*_2_. The learning rule of a *β*-model is given by

$$dW=\beta_{u}\langle \left(\tilde{x}-\langle \tilde{x}\rangle \right)\;{\left(\tilde{s}-\langle \tilde{s}\rangle \right)}^{T}\rangle +\varepsilon^{\beta },$$ (18)

where *ε* ^*β*^ is a matrix of error and its elements obey *p*(*ε* ^*β*^_*ij*_| *β*_1_,*β*_2_) = *N*(*ε* ^*β*^_*ij*_; 0, *Σ* ^*εβij*^). The negative log likelihood function for *β* is defined by

$$\begin{array}{l} L_{\beta }\;=\;-\sum_{i}{}_{,j}\text{log}N\left(\varepsilon^{\beta }{}_{ij};\;0,\Sigma^{\varepsilon \beta ij}\right)\\ =\;\frac{1}{2}\sum_{l=1}^{100}\sum_{i,j}^{}\frac{1}{2\Sigma^{\varepsilon \beta ij}}{\left\{dW_{ij}\left(l\right)\;-\;\beta_{1}\left(z_{ij}{}^{1,0}\left(l\right)\;+\;z_{ij}{}^{0,1}\left(l\right)\right)\;-\;\beta_{2}z_{ij}{}^{1,1}\left(l\right)\right\}}^{2}\;+\;50\;\sum_{i,j}\text{log}2\pi \left|\Sigma^{\varepsilon \beta ij}\right|. \end{array}$$ (19)

We also assume *Σ* ^*εβij*^s are common among all *i* and *j*. Under the assumption that (*β*_1_, *β*_2_)^*T*^ obeys *q*((*β*_1_, *β*_2_)^*T*^) = *N*((*β*_1_, *β*_2_)^*T*^; (*μ* ^*β*1^, *μ* ^*β*2^)^*T*^, *Σ* ^*β*^), the expectation of (*β*_1_, *β*_2_)^*T*^ that gives the minimum of *L*_*β*_ is given by

$$\left(\begin{array}{l} \mu^{\beta 1}\\ \mu^{\beta 2} \end{array}\right)={\left[\sum_{l=1}^{100}\sum_{i,j}^{}\left(\begin{array}{cc} {\left(z_{ij}{}^{1,0}+z_{ij}{}^{0,1}\right)}^{2} & (z_{ij}{}^{1,0}+z_{ij}{}^{0,1})z_{ij}{}^{1,1}\\ z_{ij}{}^{1,1}(z_{ij}{}^{1,0}+z_{ij}{}^{0,1}) & {\left(z_{ij}{}^{1,1}\right)}^{2} \end{array}\right)\right]}^{-1}\sum_{l=1}^{100}\sum_{i,j}^{}\left(\begin{array}{l} dW_{ij}(z_{ij}{}^{1,0}+z_{ij}{}^{0,1})\\ dW_{ij}z_{ij}{}^{1,1} \end{array}\right),$$ (20)

where *dW*_*ij*_(*l*) and *z*_*ij*_^**u**^(*l*) are simplified as *dW*_*ij*_ and *z*_*ij*_^**u**^.

## Supporting Information

S1 Movie A schematic movie of experimental procedure at trial 1–10. Setup is the same as that described in Fig 3.
(MP4)

S2 Movie A schematic movie of experimental procedure at trial 91–100. (MP4)

S3 Movie The evoked response transient of populations of cultured neurons throughout training. Axes and colors are same as those in Fig 7A and 7B.
(MP4)

S1 Dataset Summarized dataset of responses of cultured neurons. Data are composed of conditional expectation transients for each condition (x_u_trn.csv, …, x_u_alt4.csv), Kullback-Leibler divergence transients for each condition (kld_trn.csv, …, kld_alt4.csv), and trains of evoked spike number at trial 1, 11, …, 91 in each culture in the TRN group (x(t)_trn_1.csv, …, x(t)_trn_23.csv). In file names, alt1, alt2, alt3, and alt4 indicate data under the alternative conditions where (*a*, *ρ*) = (1/2, 1/2), (3/4, 1/4), (3/4, 3/4), and (1, 1/2), respectively.
(ZIP)

S1 Note Estimation of learning rule. (DOCX)

S1 FigResponse properties of cultured neurons to a mixture set of hidden sources.
**(A)** Distribution. Red circles (open and filled) are *u*_1_-preferring electrodes (*n* = 371 electrodes from 23 cultures). Blue circles (open and filled) are *u*_2_-preferring electrodes (*n* = 345 electrodes from 23 cultures). As all trial average, the response of 13.5% of *u*_1_-preferring electrodes to the **u** = (1,0) state was 3 times larger than that to the (0,1) state (filled red circles; *n* = 50 electrodes from 23 cultures). In addition, the response of 12.8% of *u*_2_-preferring electrodes to the (0,1) state was 3 times larger than that to the (1,0) state (filled blue circles; *n* = 44 from 23 cultures). A black solid line, $\bar{x_{i}{}^{0,1}}=\bar{x_{i}{}^{1,0}}$. Black dashed lines, $\bar{x_{i}{}^{0,1}}=\bar{x_{i}{}^{1,0}}\pm 0.5$. A red line, $3\cdot \bar{x_{i}{}^{0,1}}=\bar{x_{i}{}^{1,0}}$. A blue line, $\bar{x_{i}{}^{0,1}}=3\cdot \bar{x_{i}{}^{1,0}}$. **(B)** Transient. A red curve is the ratio of electrodes with 3· *x*_*i*_^0,1^(*l*) < *x*_*i*_^1,0^(*l*) to *u*_1_-preferring electrodes. A blue curve is the ratio of electrodes with *x*_*i*_^0,1^(*l*) >3· *x*_*i*_^1,0^(*l*) to *u*_2_-preferring electrodes. Both curves increased during training. Shadowed areas are S.E.M.(TIFF)

S2 FigProperties of the neural population model.
**(A)** I/O function of evoked response of the neural population model. Horizontal axis, total inputs (${\tilde{s}}_{1}+{\tilde{s}}_{2}$). Vertical axis, total outputs of neural population (${\tilde{x}}_{1}+{\tilde{x}}_{2}$). A black curve is the mean of total output for each total input. The shadowed area is the standard deviation. Total neural output is almost proportional to total input except when ${\tilde{s}}_{1}+{\tilde{s}}_{2}$ = 0, i.e., when **u** = (0,0) state. Since we assume that Hebbian plasticity does not occur when **u** = (0,0) state, effectively, we can regard the I/O function as linear for considering learning rule of neural networks. **(B)** *γ*-norm of connection strengths. Notably, we define *γ*-norm by ${\left({\left|W_{11}\right|}^{\gamma }+{\left|W_{12}\right|}^{\gamma }+{\left|W_{21}\right|}^{\gamma }+{\left|W_{22}\right|}^{\gamma }\right)}^{1/\gamma }$. Red, black, and gray curves are transients of norms with *γ* = 1, 2, and 4, respectively. The red curve gradually decreased between trial 20 and 100, while the black and gray curves maintained almost same value between trial 20 and 100. Therefore, if there is a constraint on total synaptic strength as predicted by theoretical studies [9], norm with *γ* = 2–4 is more consistent with experimental data than that with *γ* = 1.(TIFF)

S3 Fig Free energy properties in cultured neural networks in the presence of 20-μM APV. **(A)** Connection strengths of the neural population. Black circles and squares are *W*_11_ and *W*_22_. White circles and squares are *W*_12_ and *W*_21_. Bars are S.E.M. **(B)** The change in connection strengths (trial 1 vs. trial 100). In the presence of 20-μM APV, *W*_11_ and *W*_22_ increased after training (**, *p* < 10^−2^; *n* = 18 from 9 cultures), and *W*_12_ and *W*_21_ also increased (*, *p* = 0.024; *n* = 18 from 9 cultures). **(C)** Transition of the expectation of internal energy (〈*U*〉; white circles), Shannon entropy (*H*; black circles), and free energy (*F*; red circles). Bars are S.E.M. **(D)** The change in 〈*U*〉, *H*, and *F* (trial 1 vs. trial 100) in the presence of 20-μM APV. After training, the expectation of internal energy did not change (*p* = 0.250; *n* = 9 cultures), Shannon entropy slightly increased (*, *p* = 0.027; *n* = 9 cultures), and free energy did not change (*p* = 1.000; *n* = 9 cultures).
(TIFF)

S4 Fig Hebbian plasticity with a *γ*-norm constraint on total connection strength. **(A)** The expectations of *α’* and *λ* when we change the degree of *γ*-norm constraint. Black and gray curves are the mean of *μ* ^*α’*^(*γ*) and *μ* ^*λ*^(*γ*), respectively. **(B)** BIC of the *α’*-model when we change the degree of *γ*-norm constraint. A black curve is the mean of BIC. A dashed line is BIC of the *β*-model. Red, black, and gray arrows correspond to red, black, and gray curves in S2B Fig, respectively. **(C)** Bayesian model comparison between *α’*- and *β*-models. For the wide range of *γ*, the *β*-model is more plausible than the *α’*-model to represent experimental data (*, *p* = 0.035 for *γ* = 1, red circles; ****, *p* < 10^−5^ for *γ* = 2, black circles; ****, *p* < 10^−5^ for *γ* = 4, gray circles). Circle colors correspond to the arrow colors in (B). **(D)** The change in connection strengths estimated from the *α’*-model. A black curve, the mean of *W*_11_ and *W*_22_. A gray curve, the mean of *W*_12_ and *W*_21_. Solid lines, the true change. Dashed lines, the change estimated from the *β*-model, same as Fig 9C. In (A), (B), (D), shadowed areas are S.E.M.
(TIFF)
