# Supplementary material for: Cultured Cortical Neurons Can Perform Blind Source Separation According to the Free-Energy Principle
Source: PLoS Comput Biol. 2015 Dec 21;11(12):e1004643. doi: 10.1371/journal.pcbi.1004643 (PMC4686348; doi:10.1371/journal.pcbi.1004643)
Supplement: S1 Note — (DOCX) [file pcbi.1004643.s005.docx]

**S1 Note**

***Estimation of learning rule:*** Some computational models assume a constraint on total connection strength for the plasticity model [9]. As additional analysis, we consider Hebbian plasticity with a constraint on a *γ*-norm, which is represented as

*dW* = *α***_u_** 〈( – 〈〉) ( – 〈〉)*^T^*〉 + *λ* *g_γ_*(*W*) + *ε^α’^*, (21)

where *λ* *g_γ_*(*W*) term corresponds a competitive effect by the constraint, i.e., a derivative of the sum of *γ*th powers. Notably, *λ* is an amplitude and *g_γ_*(*W_ij_*) = |*W_ij_*|*^γ^*^–1^ sgn(*W_ij_*) is a non-linear function parameterized by *γ*, where *γ* is assumed to be fixed. We defined equation 21 as an *α’*-model. Other parameters are common with the *α*-model (see equation 15 and related text). As equation 21 is rewritten as *dW_ij_* = *α’* *z_ij_*^¬0,0^ + *λ* *g_γ_*(*W_ij_*) + *ε^α’^*, where *z_ij_*^¬0,0^ is the sum of *z_ij_***^u^** except (0,0) state *z_ij_*^¬0,0^ = *z_ij_*^1,0^ + *z_ij_*^0,1^ + *z_ij_*^1,1^, under the assumption that *p*(*ε^α’^_ij_*| *α’*, *λ*) is a Gaussian distribution *N*(*ε^α’^_ij_*; 0, *Σ^εα’ij^*), the negative log likelihood function for *α’* and *λ* is defined by

*L_α’_* = –∑*_i_*,*_j_* log *N*(*ε^α’^_ij_*; 0, *Σ^εα’ij^*) – *γ* log |*α*| – *γ* log |*λ*|

= $\sum_{l=1}^{100} \sum_{i,j} \frac{1}{2\Sigma^{\varepsilon\alpha'ij}}${*dW_ij_*(*l*) – *α’* *z_ij_*^¬0,0^(*l*) – *λ* *g_γ_*(*W_ij_*(*l*))}^2^

+ 50 log2*π*|*Σ^εα’ij^*| – *δ* log |*α’*| – *δ* log |*λ*|. (22)

Note that –*δ* log |*α*| and –*δ* log |*λ*| are the negative log priors, where *α* > 0 and *λ* < 0 are assumed, and *δ* is a small positive constant. Additionally, we assume *Σ^εα’ij^*s are common among all *i* and *j*. From equation 22, under the assumption that *α’* and *λ* obey a Gaussian distribution *q*(*α’*, *λ*) = *N*((*α’*, *λ*)*^T^*; (*µ^α’^*, *µ^λ^*)*^T^*, *Σ^α’λ^*), the expectation of *α’* and *λ* that gives the minimum of *L_α’_* is given by the iterative numerical calculation using the Gauss-Newton method. When we define ∇ = (∂/∂*α*, ∂/∂*λ*)*^T^*, the first-order derivative ∇*L_α’_*, the second-order derivative ∇∇*^T^L_α’_*, and the update of parameters’ expectations *d*(*µ^α’^*, *µ^λ^*)*^T^*/*dt* are represented as

∇*L_α’_* = –$\sum_{l=1}^{100} \sum_{i,j} \frac{1}{\Sigma^{\varepsilon\alpha'ij}}$(*dW_ij_* – *α’* *z_ij_*^¬0,0^ – *λ* *g_γ_*(*W_ij_*))$\left(\begin{matrix} z_{ij}^{\neg0,0} \\ g_{\gamma}\left(W_{ij}\right) \end{matrix}\right)$ – $\left(\begin{matrix} \delta/\alpha' \\ \delta/\lambda\end{matrix}\right)$,

∇∇*^T^L_α’_* = $\sum_{l=1}^{100} \sum_{i,j} \frac{1}{\Sigma^{\varepsilon\alpha'ij}}$ $\left(\begin{matrix} \left(z_{ij}^{\neg0,0}\right)^{2} & \left(z_{ij}^{\neg0,0}\right)g_{\gamma}\left(W_{ij}\right) \\ g_{\gamma}\left(W_{ij}\right)\left(z_{ij}^{\neg0,0}\right) & g_{\gamma}\left(W_{ij}\right)^{2} \end{matrix}\right)$ + $\left(\begin{matrix} \delta/\alpha'^{2} & 0 \\ 0 & \delta/\lambda^{2} \end{matrix}\right)$,

$\frac{d}{dt}\left(\begin{matrix} \mu^{\alpha'} \\ \mu^{\lambda} \end{matrix}\right)$ ∝ –(∇∇*^T^L_α’_*)^–1^ ∇*L_α’_*. (23)

Using equation 23, we calculated the maximum likelihood estimators of *α’* and *λ*.

Then, using the Bayesian model selection [47], we compared BIC between *β*-model and *α’*-model. We confirmed that Hebbian plasticity with a state-dependent efficacy (*β*-model) is plausible than that with *γ*-norm constraint (*α’*-model) to explain experimental results (S4 Fig).
