# Supplementary figures and images for: Cultured Cortical Neurons Can Perform Blind Source Separation According to the Free-Energy Principle
Source: PLoS Comput Biol. 2015 Dec 21;11(12):e1004643. doi: 10.1371/journal.pcbi.1004643 (PMC4686348; doi:10.1371/journal.pcbi.1004643)

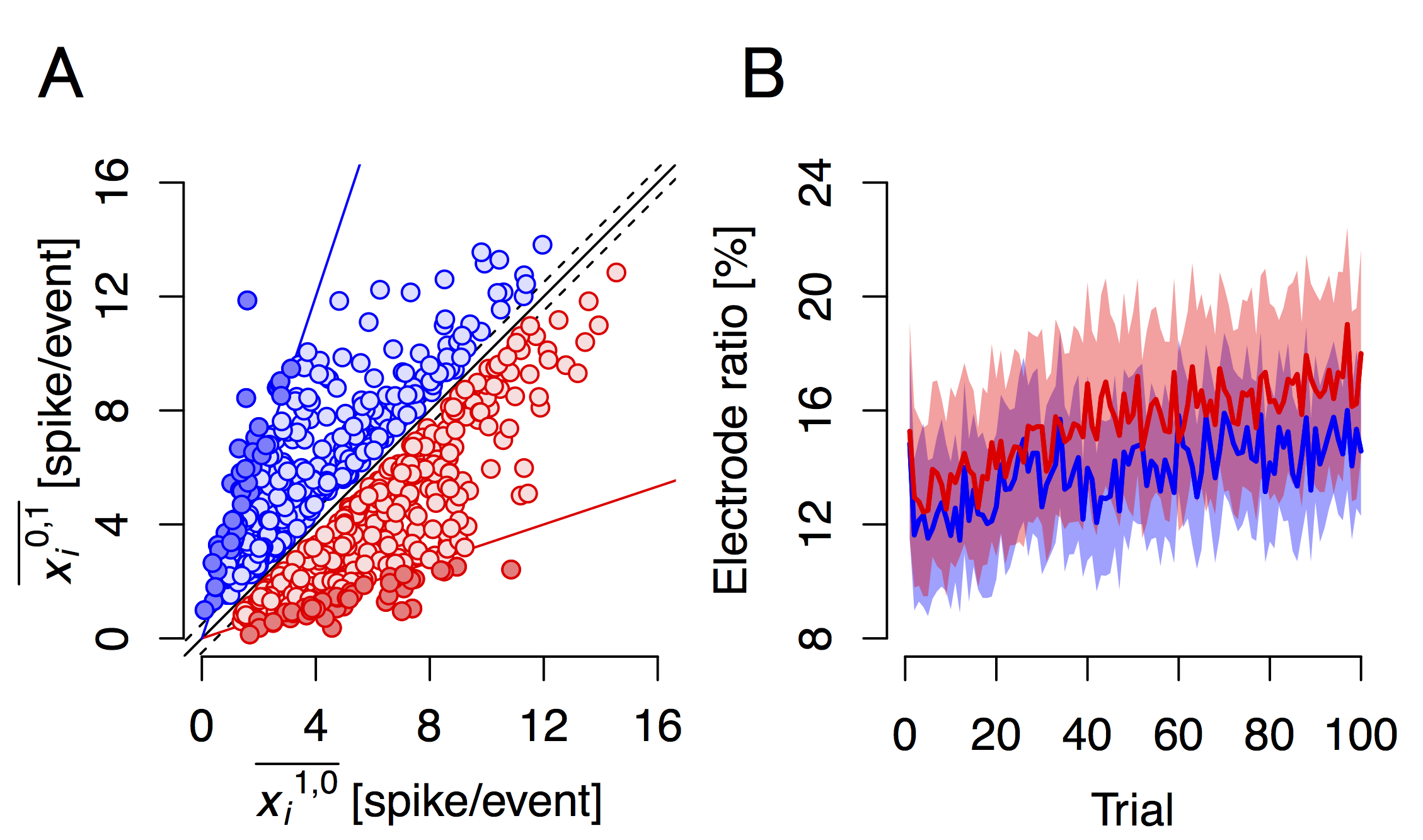

Supplement: S1 Fig — (A) Distribution. Red circles (open and filled) are u 1-preferring electrodes (n = 371 electrodes from 23 cultures). Blue circles (open and filled) are u 2-preferring electrodes (n = 345 electrodes from 23 cultures). As all trial average, the response of 13.5% of u 1-preferring electrodes to the u = (1,0) state was 3 times larger than that to the (0,1) state (filled red circles; n = 50 electrodes from 23 cultures). In addition, the response of 12.8% of u 2-preferring electrodes to the (0,1) state was 3 times larger than that to the (1,0) state (filled blue circles; n = 44 from 23 cultures). A black solid line, xi0,1¯=xi1,0¯. Black dashed lines, xi0,1¯=xi1,0¯±0.5. A red line, 3⋅xi0,1¯=xi1,0¯. A blue line, xi0,1¯=3⋅xi1,0¯. (B) Transient. A red curve is the ratio of electrodes with 3· x i 0,1(l) < x i 1,0(l) to u 1-preferring electrodes. A blue curve is the ratio of electrodes with x i 0,1(l) >3· x i 1,0(l) to u 2-preferring electrodes. Both curves increased during training. Shadowed areas are S.E.M. (TIFF) [file pcbi.1004643.s006.tiff]

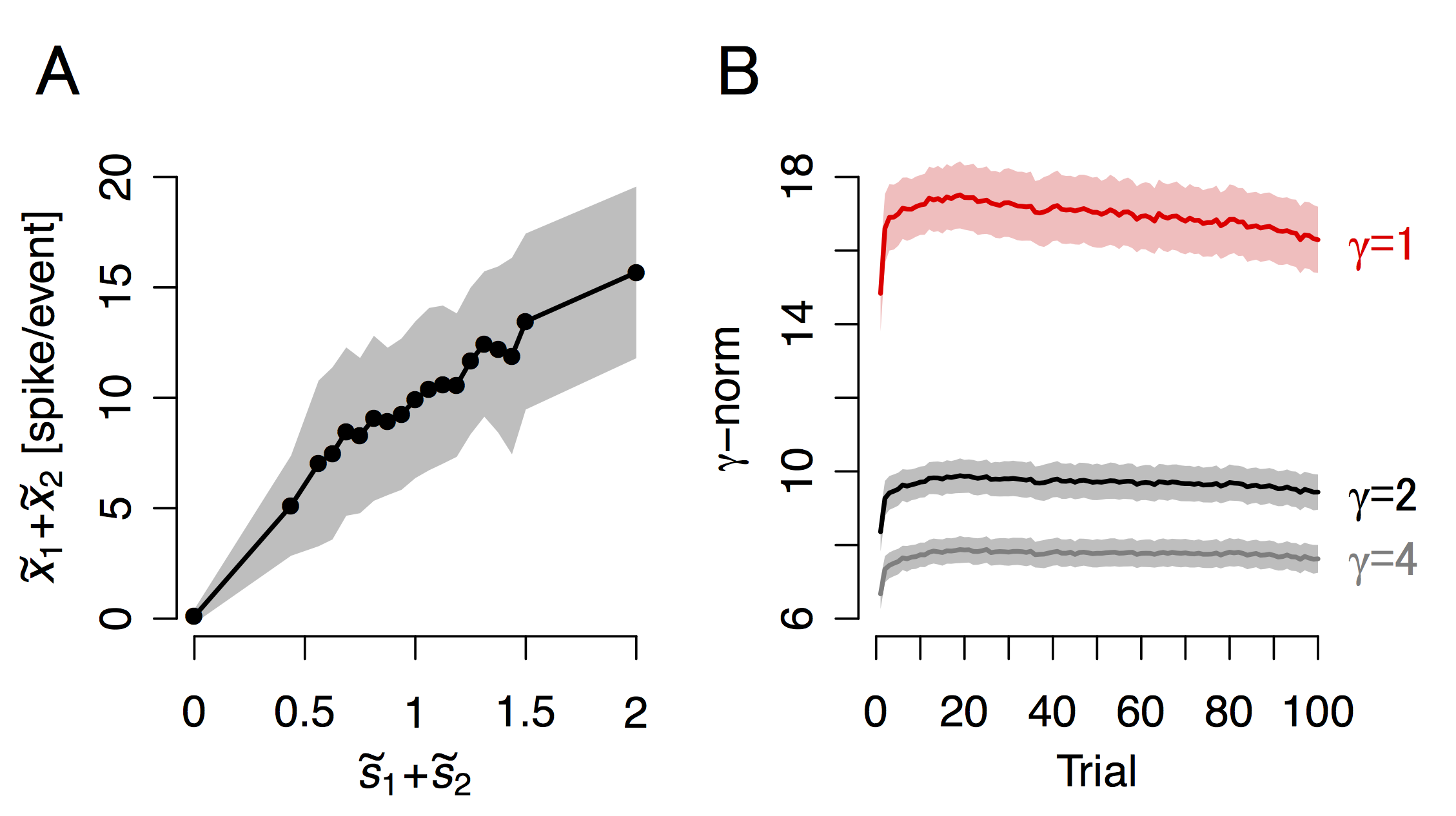

Supplement: S2 Fig — (A) I/O function of evoked response of the neural population model. Horizontal axis, total inputs (s˜1+s˜2). Vertical axis, total outputs of neural population (x˜1+x˜2). A black curve is the mean of total output for each total input. The shadowed area is the standard deviation. Total neural output is almost proportional to total input except when s˜1+s˜2 = 0, i.e., when u = (0,0) state. Since we assume that Hebbian plasticity does not occur when u = (0,0) state, effectively, we can regard the I/O function as linear for considering learning rule of neural networks. (B) γ-norm of connection strengths. Notably, we define γ-norm by (|W11|γ+|W12|γ+|W21|γ+|W22|γ)1/γ. Red, black, and gray curves are transients of norms with γ = 1, 2, and 4, respectively. The red curve gradually decreased between trial 20 and 100, while the black and gray curves maintained almost same value between trial 20 and 100. Therefore, if there is a constraint on total synaptic strength as predicted by theoretical studies [9], norm with γ = 2–4 is more consistent with experimental data than that with γ = 1. (TIFF) [file pcbi.1004643.s007.tiff]

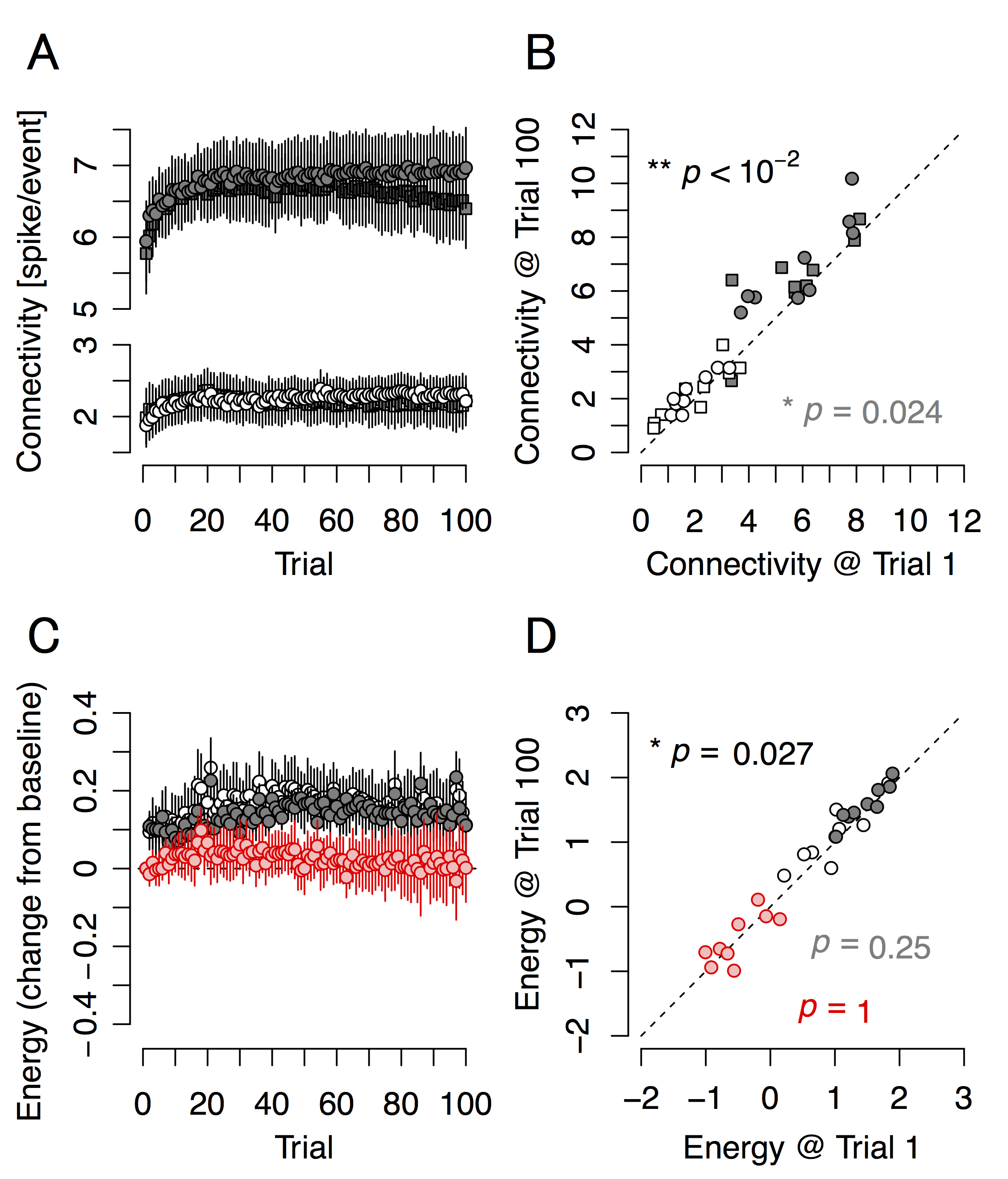

Supplement: S3 Fig — (A) Connection strengths of the neural population. Black circles and squares are W 11 and W 22. White circles and squares are W 12 and W 21. Bars are S.E.M. (B) The change in connection strengths (trial 1 vs. trial 100). In the presence of 20-μM APV, W 11 and W 22 increased after training (**, p < 10−2; n = 18 from 9 cultures), and W 12 and W 21 also increased (*, p = 0.024; n = 18 from 9 cultures). (C) Transition of the expectation of internal energy (〈U〉; white circles), Shannon entropy (H; black circles), and free energy (F; red circles). Bars are S.E.M. (D) The change in 〈U〉, H, and F (trial 1 vs. trial 100) in the presence of 20-μM APV. After training, the expectation of internal energy did not change (p = 0.250; n = 9 cultures), Shannon entropy slightly increased (*, p = 0.027; n = 9 cultures), and free energy did not change (p = 1.000; n = 9 cultures). (TIFF) [file pcbi.1004643.s008.tiff]

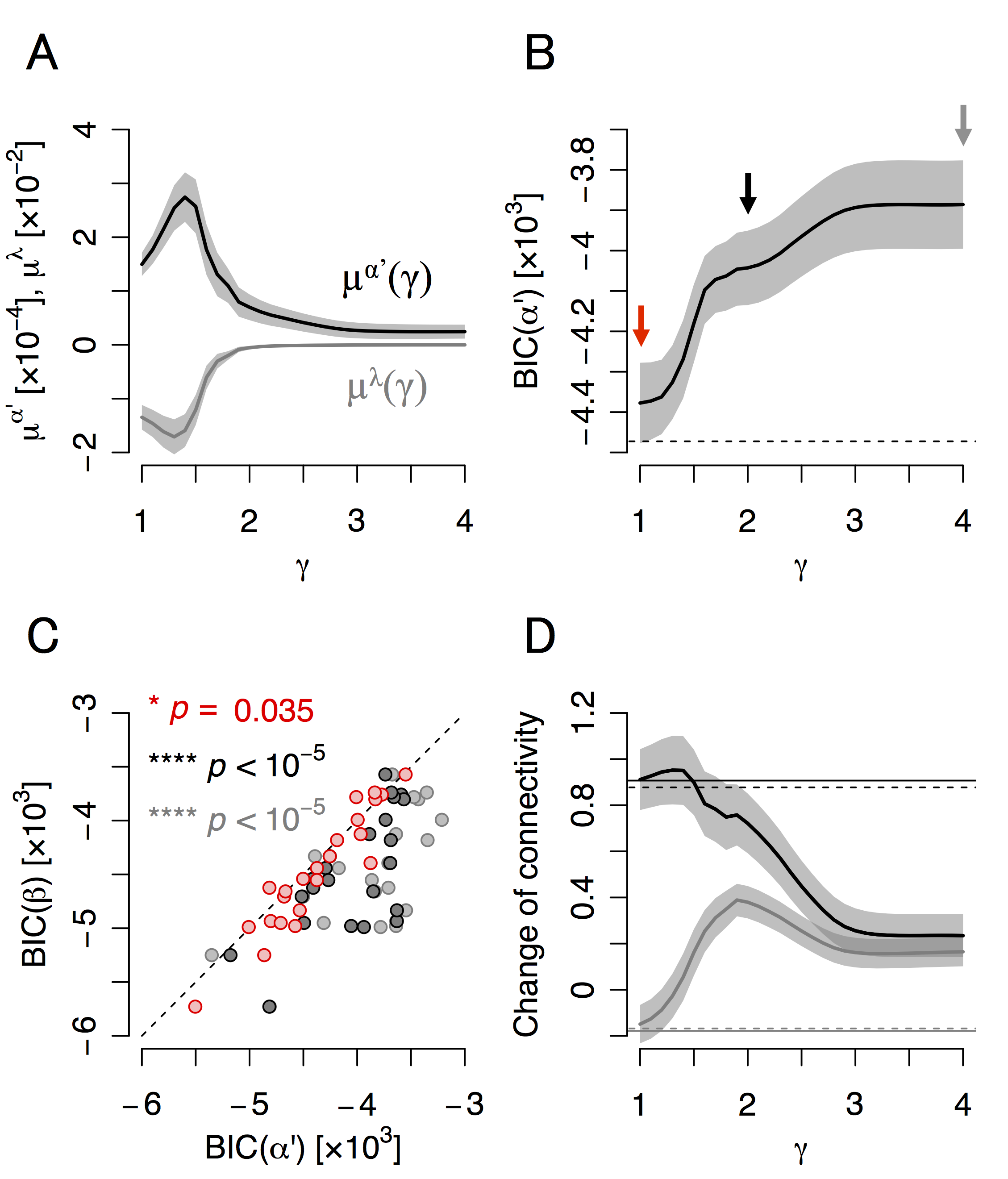

Supplement: S4 Fig — (A) The expectations of α’ and λ when we change the degree of γ-norm constraint. Black and gray curves are the mean of μ α’(γ) and μ λ(γ), respectively. (B) BIC of the α’-model when we change the degree of γ-norm constraint. A black curve is the mean of BIC. A dashed line is BIC of the β-model. Red, black, and gray arrows correspond to red, black, and gray curves in S2B Fig, respectively. (C) Bayesian model comparison between α’- and β-models. For the wide range of γ, the β-model is more plausible than the α’-model to represent experimental data (*, p = 0.035 for γ = 1, red circles; ****, p < 10−5 for γ = 2, black circles; ****, p < 10−5 for γ = 4, gray circles). Circle colors correspond to the arrow colors in (B). (D) The change in connection strengths estimated from the α’-model. A black curve, the mean of W 11 and W 22. A gray curve, the mean of W 12 and W 21. Solid lines, the true change. Dashed lines, the change estimated from the β-model, same as Fig 9C. In (A), (B), (D), shadowed areas are S.E.M. (TIFF) [file pcbi.1004643.s009.tiff]
